# Functional and Structural Analysis of a Highly-Expressed *Yersinia pestis* Small RNA following Infection of Cultured Macrophages

**DOI:** 10.1371/journal.pone.0168915

**Published:** 2016-12-28

**Authors:** Nan Li, Scott P. Hennelly, Chris J. Stubben, Sofiya Micheva-Viteva, Bin Hu, Yulin Shou, Momchilo Vuyisich, Chang-Shung Tung, Patrick S. Chain, Karissa Y. Sanbonmatsu, Elizabeth Hong-Geller

**Affiliations:** 1 Bioscience Division, Los Alamos National Laboratory, Los Alamos, New Mexico, United States of America; 2 Theoretical Division, Los Alamos National Laboratory, Los Alamos, New Mexico, United States of America; East Carolina University Brody School of Medicine, UNITED STATES

## Abstract

Non-coding small RNAs (sRNAs) are found in practically all bacterial genomes and play important roles in regulating gene expression to impact bacterial metabolism, growth, and virulence. We performed transcriptomics analysis to identify sRNAs that are differentially expressed in *Yersinia pestis* that invaded the human macrophage cell line THP-1, compared to pathogens that remained extracellular in the presence of host. Using ultra high-throughput sequencing, we identified 37 novel and 143 previously known sRNAs in *Y*. *pestis*. In particular, the sRNA Ysr170 was highly expressed in intracellular *Yersinia* and exhibited a log2 fold change ~3.6 higher levels compared to extracellular bacteria. We found that knock-down of Ysr170 expression attenuated infection efficiency in cell culture and growth rate in response to different stressors. In addition, we applied selective 2’-hydroxyl acylation analyzed by primer extension (SHAPE) analysis to determine the secondary structure of Ysr170 and observed structural changes resulting from interactions with the aminoglycoside antibiotic gentamycin and the RNA chaperone Hfq. Interestingly, gentamicin stabilized helix 4 of Ysr170, which structurally resembles the native gentamicin 16S ribosomal binding site. Finally, we modeled the tertiary structure of Ysr170 binding to gentamycin using RNA motif modeling. Integration of these experimental and structural methods can provide further insight into the design of small molecules that can inhibit function of sRNAs required for pathogen virulence.

## Introduction

*Yersinia pestis*, the etiological agent of plague, has been the cause of multiple epidemics throughout human history, and is currently classified as a select agent, signifying great potential biothreat risk for adverse impact on public health. *Y*. *pestis* is transmitted to humans through the bite of an infected flea, and has recently diverged from *Y*. *pseudotuberculosis*, a human enteropathogen that causes gastrointestinal disease through the oral route.[1] Upon host infection, pathogenic *Yersinia* induce expression of multiple virulence genes, including the Type III secretion system (T3SS), to modulate the host immune response and promote pathogen survival.[2]

Recently, bacterial small RNAs (sRNAs) have been linked to regulation of virulence in multiple pathogens[3, 4], including *Yersinia* species. Bacterial sRNAs are thought to primarily regulate gene expression by base-pairing with multiple downstream target mRNAs to attenuate translation of mRNA into protein, although other mechanisms may exist.[5] By modulating the expression levels of target genes, sRNAs can enable rapid adaptation of cellular physiology in response to environmental change.[6] Application of global analysis approaches, such as high-throughput microarray and ultra high-throughput sequencing, have begun to systematically identify bacterial sRNA populations encoded in the intergenic regions (IGRs) of the genome, generating hundreds of sRNA candidates that are actively being investigated to determine their functional activities.

Given their potential regulatory roles in virulence, sRNAs may represent an unexploited category of molecular targets for antimicrobial design. Several classic antibiotics, including aminoglycosides and tetracycline, were shown to bind ribosomal RNA and block protein synthesis decades after their initial discovery[7, 8], demonstrating the druggability of RNA. In order to assess whether sRNAs are viable candidates for antimicrobial drug discovery, several challenges remain to be addressed. First, functional roles for defined sRNAs must be validated. Second, given that bacterial sRNAs can vary in length from ~50 to 500 nucleotides and assume complex folded structures similar to proteins, experimental determination of sRNA structure is needed to predict sRNA folding. Finally, experimental and modeling methods to analyze sRNA-small molecule interactions can provide insight into the design of inhibitors that block target sRNA function.

To identify sRNAs that are likely to function in virulence, we utilized deep sequencing to compare sRNA expression in intracellular *Y*. *pestis* that invaded the human macrophage cell line THP-1 to *Y*. *pestis* that remained extracellular in the presence of THP-1 cells. We verified the expression of 143 known sRNAs and identified an additional 37 novel sRNAs in *Y*. *pestis*. We found a single *Yersinia* sRNA with transcript levels >3.5 log2 higher in the intracellular versus the extracellular fraction, whereas the fold changes for all other sRNAs were <1. Interestingly, we had shown that this same sRNA, previously designated Ysp8, was highly expressed in monoculture *Y*. *pestis* at 37°C, the temperature of the human host, compared to 26°C.[9] In a concurrent study, Beauregard et al. had identified ysr170, a 125 nt transcript that is contained within the Ysp8 sequence at the 3’ end, and reported that expression was also dependent on temperature and the presence of the RNA chaperone Hfq.[10] Since sRNA nomenclature has yet to be standardized, we will henceforth refer to Ysp8 as Ysr170 to minimize confusion. Here, we show that knock-down of Ysr170 expression can attenuate infection efficiency in cell culture and bacterial cell growth rate in response to stress, and enhance host immune response during infection. We also analyzed Ysr170 secondary structure using SHAPE analysis in the presence of small molecule antibiotics and Hfq. In the long-term, we anticipate that linking sRNA functional validation to structural analysis will enable sRNA-targeted antibiotic development by screening for small molecules that block sRNA folding or key sRNA/mRNA interactions.

## Materials and Methods

### RNA sample preparation from intracellular and extracellular *Y*. *pestis* CO92 upon infection of human monocytic THP-1 cells

We used the following bacterial strains: (1) pathogenic *Y*. *pestis* CO92 (pCD1+), (2) attenuated *Y*. *pestis* CO92 (pCD1-), and (3) pathogenic *Y*. *pseudotuberculosis* (ATCC 4284). THP-1 cells (2x10^7^) were pre-treated with 100 nM PMA 72h prior to infection, and culture media (RPMI supplemented with 10% fetal bovine serum, 10-RPMI) was replaced 48h post-PMA treatment. Overnight cultures of *Y*. *pestis* grown at 25°C and 200 rpm in Brain Heart Infusion (BHI) media were diluted 10-fold in BHI and incubated at 37°C for 2h, at 200rpm. Temperature-stimulated cultures at OD_600_ ~0.6 were used to infect THP-1 cells at multiplicity of infection (MOI) 5 for 3 hrs. The conditioned media was then collected and subjected to two sequential centrifugation steps: (1) 1,200 rpm for 2 min to remove carryover non-adherent host cells, and (2) 4,000 rpm for 10 min to pellet extracellular bacteria. A control *Y*. *pestis* culture not exposed to host cells was processed in parallel for downstream gene expression comparisons.

Extracellular bacterial pellets were treated with 0.1% Triton in 1xPBS for 2 minutes to lyse carryover host cells, resuspended in 10 ml PBS, and collected by centrifugation at 4,000 rpm for 10 min. To obtain the intracellular bacterial fraction, THP-1 cells that remained after collecting the conditioned media were washed with 20 ml PBS five times and resuspended in fresh 10-RPMI for 3 additional hours (total 6h exposure to *Y*. *pestis*). This conditioned media was then discarded, and THP-1 cells were incubated in 10 ml TES (10 mM TrisHCl, pH 7.5, 1mM EDTA, and 100 mM NaCl) buffer containing 10 mg/ml lysozyme for 30 min at 37°C and 5% CO_2_ to weaken cell wall attachment of any remaining extracellular bacteria. The THP-1 cells were then washed with PBS, incubated in 0.1% Triton for 5 minutes, and subjected to two centrifugation steps, 1,200 rpm for 2 min and 4,000 rpm for 10 min to collect bacteria associated with host cells.

The extracellular and intracellular bacterial pellets were then resuspended in 10 μl of Ready-Lyse Lysozyme solution in 200 μl of TES buffer for 15 min at room temperature. Total RNA was isolated by cell lysis in TRIZOL reagent (1ml per 10^7^ estimated bacterial cells), phenol/chloroform extraction, and isopropanol precipitation from the aqueous fraction. For the intracellular RNA fraction, a poly-A RNA removal kit was applied, which increased the percentage of bacterial versus host transcript reads to ~20%. Ribosomal RNA was depleted using a combination of Human/Mouse/Rat and Bacterial Ribo-Zero kits (Illumina, Inc.). We used three biological replicates for the intracellular and extracellular samples.

### Sequencing and sRNA discovery

RNA fractions were processed into directional cDNA libraries for 2x100 bp paired-end sequencing on the Illumina HiSeq 2000. Sequencing reads were aligned to the *Y*. *pestis* CO92 reference genome (chromosome NC_003143 and plasmids NC_003131, NC_03132, NC_003134) using Bowtie2 with default settings.[11] Data from the Illumina flow cell lanes were combined into a single BAM file for each replicate and samtools mpileup was used to output coverage files. We retrieved 421 sRNA predictions from RefSeq, the Rfam database version 12.0[12] and three previous RNA sequencing studies.[10, 13, 14] For the two studies that reported sRNA coordinates for different *Y*. *pestis* strains, KIM6[10] and 201[14], we used megablast to find matching positions on the CO92 RefSeq genome. In order to detect novel sRNAs, we used the Rockhopper system[15] and further identified potential sRNAs by sorting intergenic regions (IGR) greater than 50 bp by the maximum coverage in any 25 bp window. The sequencing files have been deposited in the NCBI Short Read Archive with the study accession SRP063571.

For each sRNA prediction, we plotted the relative coverage (reads per billion) in a 3,000 bp window and a larger 10,000 bp window to better detect expression across operons (S1 Fig). For the detection step only, we combined replicates and used a single black dotted line for control and solid red or blue lines for intracellular and extracellular conditions, respectively. We included a number of genome features in the plots including protein coding regions and pseudogenes from RefSeq, ERIC and YPAL repeats[16], computational predictions of sRNAs using SIPHT[17], and rho-independent transcription terminators using TransTermHP.[18] The CDSs are marked in green, pseudogenes in gray, repeats in yellow, putative sRNAs in red, and terminators in black.

The coverage plots were also used to categorize expression patterns including 1) peaks matching predicted coordinates, 2) peaks different from coordinates, and 3) no detected peak. The sRNAs without a detected peak were either not expressed or expressed as part of a 5’ UTR or operon with similar fold changes and expression levels as the flanking genes. We manually adjusted the coordinates of 57 sRNA predictions to fit the observed peaks and then counted reads using htseq-count to identify differentially expressed sRNAs using DESeq.[19]

### RNA analysis

#### Northern blotting

Total RNA (20 μg) was separated on a 6% denaturing polyacrylamide gel, transferred, and cross-linked onto Hybond-N+ nylon membrane (GE Healthcare, Amersham). Membranes were pre-hybridized in 15ml hybridization buffer (10% SDS, 1M sodium phosphate buffer pH 7.2 and 10 μg/ml denatured sonicated salmon sperm DNA) at 45^°^C for 30min. Biotinylated probes (Integrated DNA Technologies, S1 Table) were added to the hybridization buffer at a final concentration of 1nM and incubated at 45^°^C overnight. The next morning, membranes were washed 2 x 15min in 2x SSC, 0.1% SDS, followed by 2x 5min in 0.1x SSC, 0.1% SDS. Probe signal was detected using the Chemiluminescent Nucleic Acid Detection Module (Thermo Scientific), according to manufacturer’s instructions. Band size was estimated by comparing to a polyA-tailed RNA ladder run in parallel and detected with a biotinylated oligo dT probe (Life Technologies).

#### RACE (rapid amplification of cDNA ends)

1 μg of DNaseI-treated total RNA was circularized using RNA ligase I (NEB) and reverse-transcribed by PCR using gene-specific outward primers that hybridize to internal sequences in the sRNA (S1 Table). PCR-amplified fragments were separated on a 3% agarose gel, and fragments that were the predicted size of the target sRNA were excised and cloned into the pGEM T-easy vector (Promega). Sanger sequencing of the PCR products was performed by GenScript (GenScript USA Inc.) using the T7 primer.

#### Real-time PCR

Reverse transcription was performed using 1 μg total RNA and random primers with the RETROscript kit (Life Technologies). Quantitative real-time PCR was performed using SYBR green and gene-specific primers (S1 Table). IL-8 and EGR1 expression levels were determined by qPCR using TaqMan Gene Expression Assays (Applied Biosystems).

### Generation of *ysr170* knockdown strain

The full-length sequence of *ysr170* was cloned from total *Y*. *pestis* RNA into the pGem T-easy vector using the One-step Reverse Transcription PCR Kit (Invitrogen) (S1 Table). After confirming the sequence, the ysr170 fragment was cloned into pBluescript II SK+ using the SacII/SacI restriction sites to generate the Ysr170 knockdown (KD) vector. The KD vector and empty pGem T were electroporated into *Y*. *pestis* and *Y*. *pseudotuberculosis* to produce KD and control strains, respectively. Electrocompetent cells were generated by bacterial growth overnight at 26^°^C in BHI medium, culture dilution the following morning to OD_600_<0.1 in SOB medium, and continued growth at 26^°^C till OD_600_ reached 0.5–0.8. Bacterial cells (1x10^10^) were harvested and washed in ice-cold ddH2O (1x) and transformation buffer (2x, 272mM sucrose, 15% glycerol). Bacterial pellets were resuspended in 200 μl transformation buffer, aliquoted into 4 vials, frozen on dry ice immediately, and stored at -80°C. Vectors (500 ng) were added to electrocompetent cells on ice and then transferred to a pre-chilled 1mm electroporation cuvette (Eppendorf) on ice for 1 min. Electroporation was performed using a Gene Pulser Xcell Electroporation System (BioRad) at 1250V, 25uF, 200Ω. After a single pulse, cells were transferred to 1 ml of SOC medium to recover at 26^°^C for 2 hrs and then spread on BHI plates containing ampicillin for growth at 26^°^C.

### Host cell and pathogen assays

THP1 cells (1x10^5^, ATCC #TIB-202) were seeded in 1 ml of differentiation medium (100 ng/ml PMA in RPMI) in each well of a 24-well plate for 24 hrs to allow cells to adhere. *Y*. *pestis* or *Y*. *pseudotuberculosis* was grown overnight at 26^°^C, diluted to OD_600_<0.1 in BHI medium supplemented with 2.5 mM calcium chloride, and re-grown at 37^°^C for 2hrs. THP1 cells were infected at MOI 5 for 30min and washed with 1x PBS twice. Cells were then incubated in RPMI/10% FBS supplemented with 170 μg/ml chloramphenicol for an additional 24hrs. After washing with PBS once, THP1 cells in each well were lysed with 500μl lysis buffer (0.1% TritonX-100 in PBS) at 37^°^C for 10min with periodic vigorous pipetting. Then 500 μl BHI medium was added to each well and bacteria were spread on BHI agar plates in serial dilution. Plates were grown at 26^°^C for 48hrs and colonies were counted.

For ELISA measurements, THP1 cells (1x10^6^) were plated in each well of a 6-well plate in 2ml RPMI/10% FBS the day before infection. Bacteria were grown overnight at 26^°^C, diluted to OD_600_<0.1 in BHI medium supplemented with 2.5mM calcium chloride, and re-grown at 37^°^C for 2hrs. THP1 cells were infected at MOI~5. Chloramphenicol was added to a final concentration of 170μg/ml at 2hrs post-infection. Supernatants were collected at 24hrs post-infection, and ELISAs was performed using a TNFα ELISA kit (BD Biosciences) according to manufacturer’s instructions.

### Growth curves

KD and control colonies of *Y*. *pestis* and *Y*. *pseudotuberculosis* were grown overnight at 26^°^C in BHI medium, with ampicillin when appropriate. The next morning, bacteria were diluted with BHI medium (with ampicillin when appropriate) till OD_600_<0.2 and re-grown at 37^°^C. The OD_600_ was measured every 2hrs. For stress stimulations, overnight cultures were diluted with LB medium containing one of the following supplements, and re-grown at 37^°^C: pH 5.5 for mild acid stress, 10% FBS for serum stress, 100μM 2,2’-dipyridyl for iron starvation, and 1mM H_2_O_2_ for oxidative stress.

### Protein expression of Hfq

The gene coding for the Hfq protein was amplified from the chromosomal DNA of *Y*. *pestis* by PCR. (S1 Table) The primers contained restriction sites for cloning into plasmid pQE80L (Qiagen), making use of the T7 promoter and 6xHis tag for expression and purification. The resulting vector (pQE-Hfq) was transformed into *E*. *coli* BL21 (DE3) cells for overexpression. Cells were grown to OD_600_ ~0.6 at 37°C while shaking, and expression was induced by the addition of 1 mM IPTG (Fisher Scientific). Cultures were then incubated for 2 hours before harvesting. Cells were lysed and prepared for Hfq purification as described.[20] Hfq was purified using Ni-NTA affinity chromatography (Histrap FF, GE Lifesciences) on an AKTA explorer (GE Lifesciences). Lysate was loaded in buffer A (50 mM NaH_2_PO4, 300 mM NaCl, 10 mM imidazole) and Hfq was then eluted by applying a gradient to 100% buffer B (50 mM NaH_2_PO4, 300 mM NaCl, 0.5 M imidazole) over 25 column volumes. Peaks were collected, and fractions containing Hfq were verified by SDS-PAGE electrophoresis. Samples were concentrated and buffers exchanged using centrifugal ultrafiltration (Amicon ultra-15, 3 kDa MWCO). Hfq was then stored in 1X HMK (50 mM HEPES pH 8.0, 100 mM KCl, 2 mM MgCl_2_) and 50% glycerol. The Hfq concentration was determined by Bradford assay (BioRad). Interactions between Hfq and sRNA were followed using both SHAPE and hydroxyl radical probing. After folding of RNA samples, bulk yeast tRNA (Ambion) was first added (0.4 μg/μl), followed by Hfq. Samples were incubated at 37°C for 10 minutes and then probed as described below.

### RNA preparation

RNA for *in vitro* analysis was transcribed from synthetic templates. The dsDNA templates for Ysr170 were prepared by overlap PCR of synthetic DNA Ultramers (IDTDNA). The sequence of the template was: 5’- AAT AAT ACG ACT CAC TAT A**GG CGA TCC CAG GTG** TAT AGT TAG CGG ATT ACT TTA TCT CAA GAA GAT AGG AGT CAT ATT ATG ACT AAA AAT ACT GCG ACT AAA GTA AAA AGC ATC AAA CTC GTA ACC TTT GGC AAA AAT ACG GCT CTG GCG GGC GCA GTG CCT AGA ACA TTA TCA GGC CAA GAA GCC GGC CGT GTG CTT GGG TTT GGG TGT CAT CAC TCT TAA TTT AAG CAT TTA ATA TAC TGG ACG TGA CCT AGA AGT AGA TAT TGG AAT AAT TTG TTC CAA TAA TCT TCA CAT AAC TTA GTG ACT CAA GCC GGG AGT GGC GGT CTT CTC ACA CCC GGC TTT TAT **GAT TCT TGT GGC ATG CTCT** -3’. The sequence includes a T7 promoter (underlined) and nucleotides (bold) at the 5’ and 3’ termini, which were added to the sequence of *ysr170* to improve primer extension reads and provide a 3’ primer binding site. The templates were transcribed with T7 RNA polymerase using the Cellscript high yield transcription kit (Epicentre) as per the manufacturer’s protocol. Samples were then treated with 1 U of DNase I for 20 min at 37°C and precipitated on ice by the addition of 1 volume of 7M ammonium acetate. Following centrifugation, the RNA pellet was lyophilized and resuspended in H_2_O. The homogeneity of the RNA was verified by PAGE (10% polyacrylamide, 7M urea, 0.5x TBE).

### EMSA analysis of Hfq interactions with Ysr170

Ysr170 was combined with an equivalent amount of Alexa-488 labeled oligo complementary to the 3’ primer binding site (AGAGCATGCCAC) and was diluted in H_2_O to 110 nM. The probe was annealed via RNA denaturation at 90°C for 2 min and snap cooled on ice for 5 min. The samples were then incubated in 1X HMK buffer (50 mM HEPES pH 8.0, 100 mM KCl, 2 mM MgCl_2_) at 37°C for 10 min to allow for RNA folding. Yeast tRNA (5 μg) was added to block non-specific interactions, followed by incubation at 37°C for 10 min with Hfq. The final reactions consisted of 80 nM Ysr170 and Hfq at 0, 0.1, 0.5, and 1 μM. Concentrations of Hfq were determined by the MW of the hexameric form. The samples were then resuspended in glycerol at a final concentration of 10%, loaded on a 6% polyacrylamide gel (0.5X TBE), and electrophoresed at 20 V/cm for 80 min. Resulting gels were scanned on a Hitachi FMBio III using 488 nm laser excitation and 530 nm emission filters.

### Structure probing

The 2’ hydroxyl acylating reagent 1M7 (1-methyl-7-nitroisatoic anhydride) was synthesized as described.[21] RNA samples were folded by heating to 95°C for 2 min in H_2_O followed by a 2 min incubation on ice. HMK buffer was then added and the RNA (final concentration, 0.5 μM) was equilibrated at 37°C for 10 min and cooled to 25°C. A one-tenth volume of 1M7 (30 mM in DMSO) was added to the sample and the reaction was incubated for 5 min at 25°C. RNA was then precipitated by the addition of 3 volumes absolute ethanol, one-tenth volume 3 M sodium acetate and 25 μg glycogen (Ambion), followed by centrifugation. RNA was dissolved in 15 μl (1μM RNA) primer extension mix containing 250 μM dNTPs, 3 pmoles of 5’-Alexa-488 labeled primer, in the supplied buffer and reverse transcriptase (200 U superscript III MMLV-RT from Invitrogen). Reactions were incubated at 50°C for 1 hour. Sequencing reactions were performed on unmodified RNA in the same manner, but the mix was supplemented to 100 μM with one of the four ddNTPs. Primer extension reactions were loaded onto a P-6 micro-biospin column (Bio-Rad) and centrifuged to remove salt and nucleotides. The samples were then lyophilized and resuspended in highly deionized (Hi-Di) formamide for analysis. Each sample was diluted 1:20 in Hi-Di formamide and heated to 95°C for 2 min. The samples were electrokinetically injected (5 s at 6 kV) onto an ABI Prism 3100 Avant quad-capillary instrument and a fluorescence electropherogram was collected (60 minutes at 14 kV). The data was then integrated and aligned using an in-house program for the simultaneous fitting of multiple Gaussian peaks to the traces. Areas were then assigned to nucleotides based on dideoxy-sequencing data and normalized between runs. Experiments were repeated a minimum of 3 times for each condition.

Hydroxyl radical probing reactions were carried in 1X HMK buffer. Fe:EDTA reactions were designed to produce a burst of hydroxyl radicals by limiting the concentration of H_2_O_2_. Reactions of 0.3 μM RNA were folded as described above followed by addition of 3% (v/v) H_2_O_2_ in 1X HMK to a final concentration of 0.03%. The mixture was then transferred to a tube containing Fe:EDTA (40.5 mM FeSO_4_, 42.5 mM EDTA) and 208 mM ascorbate in 1X HMK. After mixing, the final concentration of Fe:EDTA was 400 μM and the reactions were incubated at 25°C for 2 min, stopped by the addition of 3 volumes EtOH and 50 μg glycogen, and precipitated before reverse transcription and capillary electrophoresis.

For CMCT probing reactions, 1/10 volume of a 0.5 M 1-cyclohexyl-(2-morpholinoethyl)carbodiimide metho-p-toluene sulfonate (Sigma-Aldrich) in H_2_O was added to the folded RNA and the reaction was incubated for 20 min at 22°C. Treated RNAs were then precipitated by the addition of 1/10^th^ volume 3M sodium acetate (pH 6.5) and 3 volumes ethanol. Chemical modification reactions with dimethylsulfate (DMS, Sigma-Aldrich) were performed in 1X HMK buffer. The RNA was first folded and incubated in buffer for 10 minutes at 37°C. The reaction was initiated by the addition at a 1:100 ratio of a 10% solution of DMS in EtOH. After incubation at 25°C for 10 minutes, alkylation was stopped by the addition of 1 volume of stop buffer (1 M Tris-HCL pH 7.5, 1 M β-mercaptoethanol and 1M sodium acetate). The mixture was then precipitated with 2.5 volumes EtOH in preparation for reverse transcription and capillary electrophoresis.

### Structural modeling

A three-dimensional structural model was constructed for the region of Ysr170 shown to interact with gentamicin using our in-house RNA motif library based on available RNA crystallographic structures in the protein databank. The antibiotic binding region from the ribosome, specifically protein databank entry 2ESI, was homologous to Ysr170 and used in the models. Our RNA homology methodology has been described previously.[22]

## Results

### Deep sequencing of *Y*. *pestis* transcriptome and sequence analysis of sRNA candidates

We isolated and processed total RNA from intracellular pathogenic *Y*. *pestis* CO92 that invaded THP-1 cells and *Y*. *pestis* that remained in the extracellular medium, in order to analyze differential expression between the two populations. All together, 899 million reads were sequenced and 464 million reads were aligned to the *Y*. *pestis* CO92 reference genome. Overall, 68% of the reads from the extracellular and 11% from the intracellular pathogen fractions mapped to the reference genome. We compared the 421 sRNA predictions in RefSeq, Rfam and three previous RNA sequencing studies[10, 13, 14] using coverage plots (S1 Fig). We merged 96 duplicate predictions like sR007 and ysr151 that match the highly expressed RNaseP (Table 1). Of the remaining 325 unique predictions, we identified 143 sRNAs with a distinct peak in our sequencing data and adjusted the coordinates of 57 of these sRNAs prior to read counting (S2 Table). Another 139 sRNAs were not detected and an additional 43 were likely expressed within an operon or 5’ UTR and lacked a distinct peak in the expression plots. We also discovered another 37 novel sRNAs on the chromosome, bringing the total of sRNAs analyzed in our study to 180 (143 previously-identified and 37 novel sRNAs, S2 Table). Following the *ysr* (for *Yersinia* small RNA) numbering system in previous studies[13, 23], we designated these novel sRNAs as Ysr252 to Ysr288.

**Table 1:** List of small RNAs with mean normalized counts > 20000 in the intracellular fraction.

IDs	Start	End	Strand	Extracellular	Intracellular	Fold Change
YPOs07/RNaseP/sR007/Ysr151	3955749	3956125	-	691907	551206	-0.33
CsrC/sR026/Ysr184/Ysr188	27658	28043	+	286428	359960	0.33
YPOs02/6S/sR017/Ysr182	1001993	1002174	+	106513	130705	0.30
YPOs03/CsrB/sR003/Ysr179	1177074	1177355	+	311513	129602	-1.27
Ysr172	1973419	1973641	-	85470	59833	-0.51
Ysr271	2449900	2450155	-	71198	52815	-0.43
Ysr170	2265980	2266341	-	4069	48609	3.58
YPOs04/tmRNA/sR022	1245588	1245951	+	30943	36622	0.24
sR064/Ysr196	3815763	3815985	-	30499	27988	-0.12
Ysr286	4343211	4343317	-	41656	24912	-0.74
GcvB/sR013/ysr45/Ysr180	1169590	1169794	-	57949	22334	-1.38

**Fig 1:**
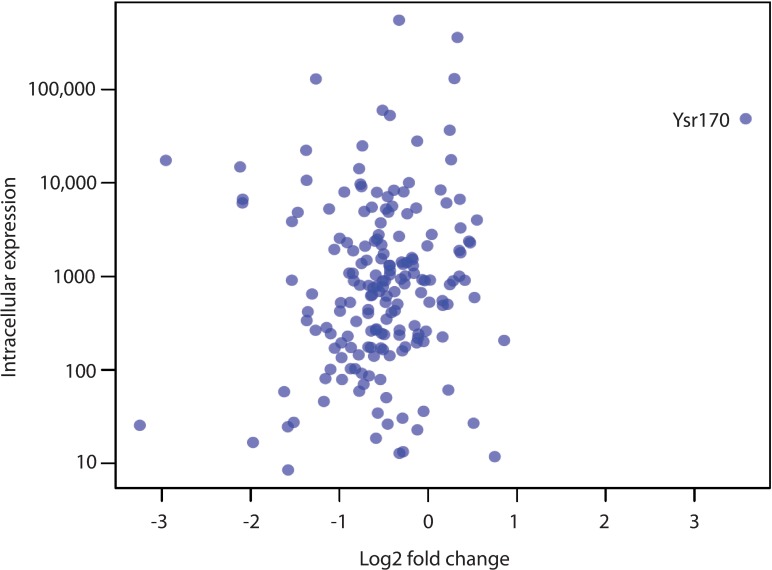
Comparison of the log2 fold change from extracellular to intracellular *Yersinia* and the mean normalized counts in intracellular *Yersinia* for all 180 small RNAs.

Although many sRNAs were highly expressed in both the intracellular and extracellular pathogen fractions (Table 1), very few sRNAs were differentially expressed (S2 Table). However, one sRNA, Ysr170, was a clear outlier, exhibiting a log2 fold change ~3.6 for intracellular expression, compared to fold changes of <1 for the other 179 sRNAs. (Fig 1 and Table 1)

### Validation of *Yersinia* sRNAs

**Fig 2:**
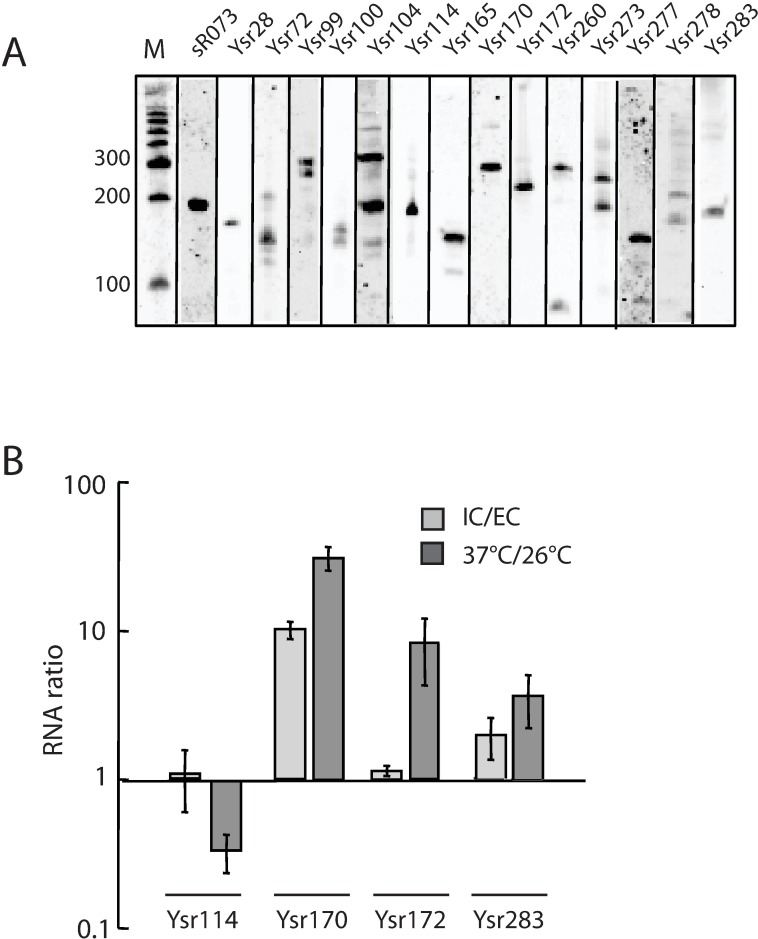
Validation of candidate sRNA gene expression. (A) sRNA expression was analyzed by northern blot. Total *Y*. *pestis* RNA was separated on a 6% denaturing polyacrylamide gel, transferred to a nylon membrane, and probed with biotinylated oligos to target specific sRNAs. The size of the transcripts was determined by using a polyA-tailed RNA ladder. (B) RT-PCR analysis was performed on total RNA isolated from *Y*. *pestis* in the intracellular (IC) or extracellular (EC) fraction after infection of THP-1 cells or grown at 37° or 26°C. The relative sRNA levels are presented as fold expression between either intracellular and extracellular fractions or the two temperature conditions. For each sample, the sRNA levels were normalized to 5S rRNA. The average mean and standard deviation from three representative experiments are shown.

We selected 15 sRNA candidates, including 5 novel sRNAs, for validation of relative size, strand, and expression levels by northern blot. (Fig 2 and Table 2) Interestingly, some of the sRNAs (e.g. Ysr99 and Ysr104) migrated as multiple bands, suggesting that they may undergo processing. Using rapid amplification of cDNA ends (RACE), we determined the exact 5' and 3' ends, and thus the genomic coordinates, for Ysr72, Ysr114, Ysr165, Ysr170, and Ysr283 (Table 2). The Ysr170 transcript was 362 nucleotides in length (coordinates 2265980–2266341) by RACE analysis, which includes the shorter 125 nucleotide Ysr170 sequence also identified via RACE in KIM6+ at the 3’ end.[10] By qRT-PCR, Ysr170 displayed ~10-fold increased expression in intracellular (IC) *Y*. *pestis* that invaded THP-1 cells compared to the extracellular (EC) fraction, consistent with the deep sequencing results. (Fig 2B) We also demonstrated that Ysr170, Ysr172, and Ysr283 were upregulated ~35, ~9, and ~3-fold at the human host temperature of 37°C compared to the flea host temperature of 26°C, whereas Ysr114 exhibited a ~50-fold higher expression at 26°C. In a previous RNAseq study based on *Y*. *pestis* growth at different temperatures, we had found that Ysr170 was expressed at higher levels at 37°C compared to 26°C.[9]

**Table 2:** List of validated sRNAs in this study.

IDs	Start	End	Strand	Extracellular	Intracellular	Fold Change	RACE
sR035/Ysr104	472556	472924	+	1485	1894	0.35	
sR073	2027880	2028036	-	492	550	0.16	
Ysr28	4375368	4375649	-	1605	648	-1.31	
Ysr72	2348534	2348614	+	977	630	-0.63	yes
Ysr99	306208	306410	+	2637	909	-1.54	
Ysr100	324968	325122	+	NA	NA		
Ysr114	1543770	1543972	+	5102	2556	-1.00	yes
Ysr165	1846560	1846676	+	689	817	0.24	yes
Ysr170	2266341	2265980	-	4069	48609	3.58	yes
Ysr172	1973419	1973641	-	85470	59833	-0.51	
Ysr260	1190114	1190273	+	201	225	0.16	
Ysr273	2667315	2667437	-	4047	1944	-1.06	
Ysr277	3193126	3193244	+	1255	897	-0.49	
Ysr278	3496394	3496544	+	870	337	-1.37	
Ysr283	4122174	4122369	+	14798	17702	0.26	yes

For Ysr72, Ysr114, Ysr165, Ysr170, and Ysr283, the Start and End coordinates reflect the results of RACE analysis. All other coordinates were estimated based on sequence analysis.

### Functional studies of Ysr170

**Fig 3:**
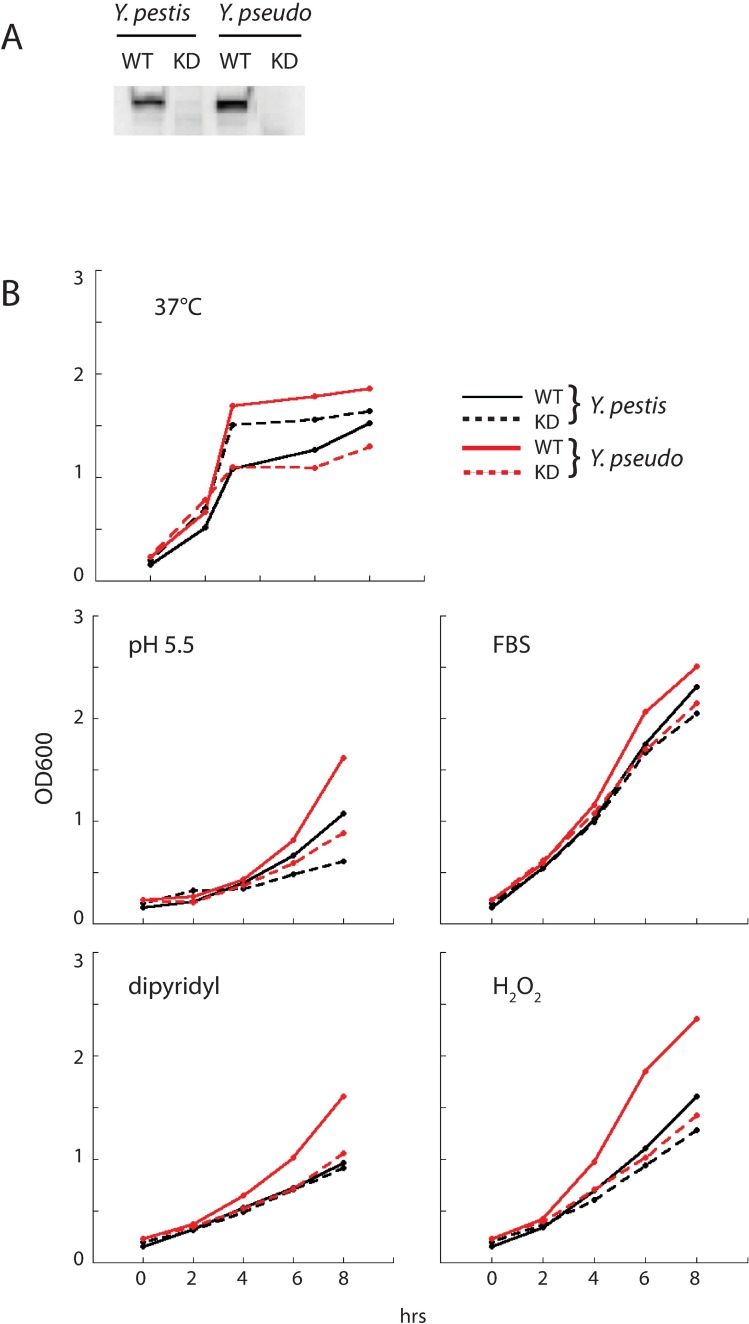
Analysis of KD Ysr170 strain. (A) Total RNA was isolated from wild-type (WT) and knock-down (KD) strains of Ysr170 in *Y*. *pestis* and *Y*. *pseudotuberculosis*, and Ysr170 expression was analyzed by northern blot. (B) Growth curves of *Y*. *pestis* (black lines) and *Y*. *pseudotuberculosis* (red lines) wild-type (solid) and Ysr170 KD (red) strains were examined in response to different stress conditions up to 8 hrs at 2 hr intervals. Stress conditions include temperature (37°C), mild acidity (pH 5.5), host serum (10% FBS), iron starvation (100μM 2,2’-dipyridyl), and oxidative stress (1mM H_2_O_2_). A representative experiment from three independent experiments is shown.

Given that Ysr170 exhibited high levels of expression under two host conditions, invasion of THP-1 cells and growth at 37°C, we further examined Ysr170 function in *Yersinia* growth, cell culture infection, and host response. Based on the genomic coordinates of Ysr170 by RACE, we generated and introduced a plasmid expressing the antisense strand of Ysr170 to produce a knockdown (KD) phenotype in both *Y*. *pestis* and *Y*. *pseudotuberculosis*. (Fig 3A) The *ysr170* sequence is 100% conserved between *Y*. *pestis* and *Y*. *pseudotuberculosis*. By northern blot, we observed that Ysr170 levels were downregulated in the KD strain compared to wild-type (WT) in both *Y*. *pestis* and *Y*. *pseudotuberculosis* grown at 37°C. (Fig 3A)

We assessed growth of the KD Ysr170 strains at 37°C and in response to several stressors that bacteria may be exposed to during host infection, including an acidic environment (pH 5.5) found in the lysosomal compartment, addition of serum (10% FBS), iron starvation (100 μM 2,2’dipyridyl), and oxidative stress (1mM H_2_O_2_) elicited by host phagocytes as a first line of defense against intracellular pathogens. (Fig 3B) Only low pH inhibited growth of the KD strain by ~2 fold in *Y*. *pestis*, indicating poor adaptation to a hostile low pH environment, such as in the lysosomes. The KD strain of *Y*. *pseudotuberculosis* exhibited a range of defective growth rates compared to the wild-type strain in response to low pH, iron starvation, and oxidative stress. Given that regulatory RNAs (e.g. sRNAs in bacteria, miRNAs in eukaryotes) have been shown to modulate expression of multiple downstream mRNAs, it is likely that Ysr170 targets multiple mRNAs that regulate bacterial cell growth in response to different stress conditions.

**Fig 4:**
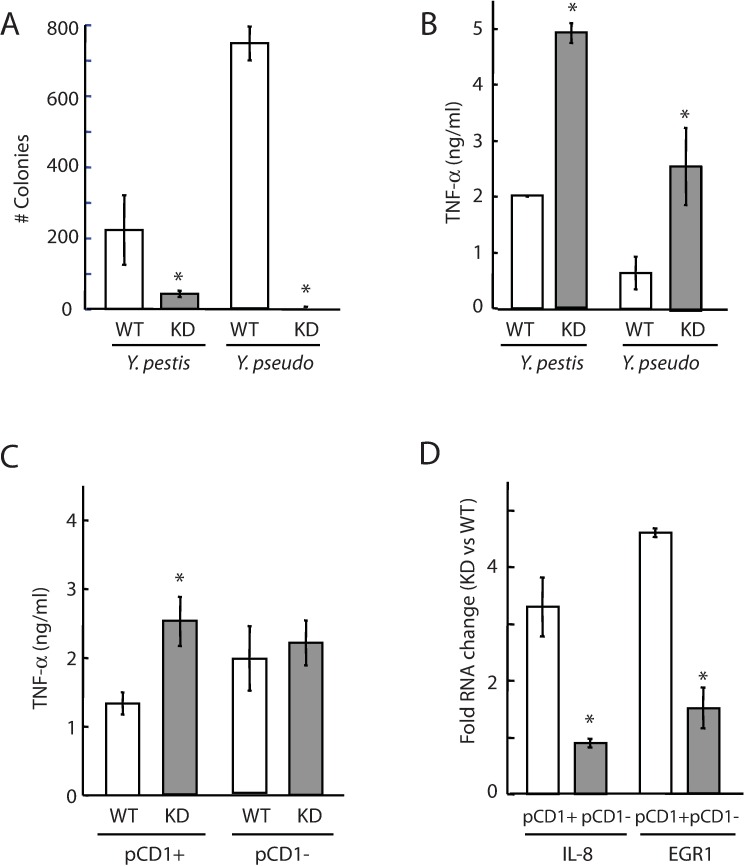
Infection efficiency of Ysr170 KD strains. (A) THP-1 cells were infected with *Yersinia* wild-type or KD Ysr170 strains at MOI 5 for 30 min to allow for pathogen invasion. Serial dilutions of THP-1 lysates (1:100) were spread on BHI agar plates, and colonies were counted after 48 hrs. The average mean and standard deviation from three representative experiments are shown. The ‘*’ denotes statistical significance (p<0.05) for number of colonies in the Ysr170 KD strains compared to wild-type (WT). (B) THP-1 cells were infected at MOI 5 with *Y*. *pestis* and *Y*. *pseudotuberculosis* wild-type or KD Ysr170 strains. After 24 hrs, cell supernatants were collected and analyzed by ELISA. The average mean and standard deviation from three representative experiments are shown. The ‘*’ denotes statistical significance (p<0.05) for TNF-α release in the Ysr170 KD strains compared to wild-type (WT). (C) THP-1 cells were infected at MOI 5 with *Y*. *pestis* pCD1+ and pCD1- strains that expressed wild-type or KD Ysr170. After 24 hrs, cell supernatants were collected and analyzed by ELISA. The average mean and standard deviation from three representative experiments are shown. The ‘*’ denotes statistical significance (p<0.05) for TNF-α release in the Ysr170 KD *Y*. *pestis* pCD1+ strains compared to wild-type (WT). (D) THP-1 cells were infected at MOI 4 with *Y*. *pestis* pCD1+ and pCD1- strains that expressed wild-type or KD Ysr170. After 24 hrs, total RNA was extracted and RT-PCR analysis was performed using specific probes to IL-8 and EGR1. The relative RNA levels are presented as fold expression in the KD Ysr170 strains compared to wild-type. For each sample, the RNA levels were normalized to 5S rRNA. The ‘*’ denotes statistical significance (p<0.05) for fold RNA change in IL-8 and EGR1 expression between pCD1+ and pCD1- strains. The average mean and standard deviation from three representative experiments are shown.

We also examined whether the Ysr170 KD strains were defective in infection of host cell culture. Both *Y*. *pseudotuberculosis* and *Y*. *pestis* Ysr170 KD strains demonstrated reduced survival within THP-1 cells (Fig 4A). There was a ~6-fold decrease in the number of *Y*. *pestis* KD colonies recovered from the host compared to wild-type *Y*. *pestis*. For *Y*. *pseudotuberculosis*, no KD colonies were recovered compared to the wild-type. These results indicate that knock-down of Ysr170 levels can attenuate bacterial intracellular survival.

To investigate the effect of Ysr170 on host immune response to *Yersinia* infection, we examined TNF-α, IL-8, and transcription factor EGR1 expression in THP-1 cells infected with wild-type and Ysr170 KD strains. In a previous study, we had demonstrated that pathogenic *Yersinia* species employ the virulence plasmid pCD1 to inhibit EGR1 and pro-inflammatory cytokine gene expression.[24] KD strains of *Y*. *pseudotuberculosis* and *Y*. *pestis* stimulated higher TNF-α cytokine release during infection of THP1 cells, compared to the Ysr170 wild-type strain (Fig 4B), and this increase was dependent on pCD1 (Fig 4C), suggesting that modulation of Ysr170 expression can attenuate *Yersinia* pCD1-driven suppression of the host immune response.[25, 26] This stronger innate immune response to *Yersinia* KD strains correlated with reduced intracellular *Yersinia* survival. (Fig 4A) Furthermore, we demonstrated that both IL-8 and EGR1 transcript levels are ~3-4-fold higher in THP-1 cells infected with KD Ysr170 strains compared to cells infected with wild-type *Y*. *pestis*. (Fig 4D) Given that pro-inflammatory cytokine and EGR1 expression in macrophages are suppressed by pCD1 [25, 27], we tested whether the KD strain of *Y*. *pestis* cured of pCD1 can alter IL-8/EGR1 expression levels, in addition to TNF-α production. We observed that IL-8 and EGR1 levels was comparable between THP-1 cells infected with KD Ysr170 *Y*. *pestis* cured of pCD1 and the pCD1-cured parental WT *Y*. *pestis* (Fig 4D), suggesting that Ysr170 may regulate expression of key virulence genes on the pCD1 plasmid to modulate host response to infection.

### Secondary structure analysis of Ysr170 by SHAPE

We have applied selective 2’ hydroxyl acylation by primer extension (SHAPE) to determine the RNA backbone flexibility and secondary structure of the Ysr170 sequence. In SHAPE, hydroxyl-selective electrophiles are used to differentially react with the 2’-OH groups of ribose moieties. Single-stranded (unconstrained) nucleotides exhibit enhanced nucleophilic reactivity, whereas base-paired (constrained) nucleotides display reduced reactivity. Following electrophoretic fragment separation, we determined helical and non-helical sub-structures to reconstruct the secondary structure of Ysr170.

**Fig 5:**
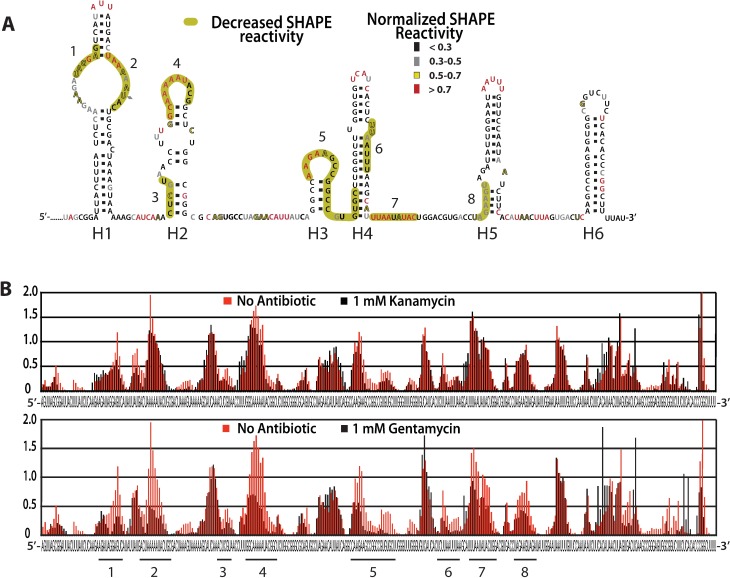
Interactions of aminoglycosides with Ysr170 were investigated using SHAPE probing. (A) 500 nM sRNA was titrated with 1mM of antibiotic. Nucleotides with high SHAPE reactivity are shown in red and nucleotides with low reactivity are shown in black. Helix 1 (H1) contains 4 internal loops/bulges and is capped by a AUU triloop with UA neck base pair. Helix 2 (H2) contains a large internal loop/bulge, a single adenine bulge, and is capped by a 9-nucleotide loop. Helices 3 (H3) and 6 (H4) contain no internal loops and are both capped by 8-member loops (note that helix 6 contains a single AA bulge). Helix 4 (H4) contains a 1–2 bulge and is capped by a tetraloop. Finally, helix 5 (H5) contains a 4–2 internal loop and is capped by a pentaloop. Proposed secondary structure showing regions with reduced SHAPE reactivity in the presence of gentamycin is shown in gold. (B) Comparison of normalized SHAPE reactivity for Ysr170 between reactions in the presence (black) or absence (red) of kanamycin (upper) or gentamycin (lower).

We found Ysr170 to be highly structured and organized into 6 extended helices containing secondary structure motifs commonly found in other structural RNAs such as the ribosome (Fig 5A). To determine how small molecule binding can potentially affect RNA secondary structure, we titrated Ysr170 with a panel of six antibiotics, including gentamicin, kanamycin, streptomycin, tetracycline, spectinomycin, and chloramphenicol. Of these six, only gentamicin significantly induced decreased SHAPE reactivity and changed Ysr170 secondary structure (Fig 5A, green regions 1–8 corresponding to Fig 5B, black lines 1–8 at bottom of figure). Interestingly, the binding of gentamicin stabilizes helix 4 (H4), which contains a bulge that structurally resembles the gentamicin binding site in the 16S bacterial ribosomal subunit. Incubation of Ysr170 with kanamycin, which lacks three methyl groups found in gentamycin, resulted in no significant changes in the overall RNA secondary structure, except for slight reactivity with helix 2 (H2). (Fig 5B top) These data illustrate a high specificity of small molecule binding to RNA secondary structure, which can elicit structural changes in RNA structure and may provide a basis for inhibitor design to disrupt sRNA folding.

**Fig 6:**
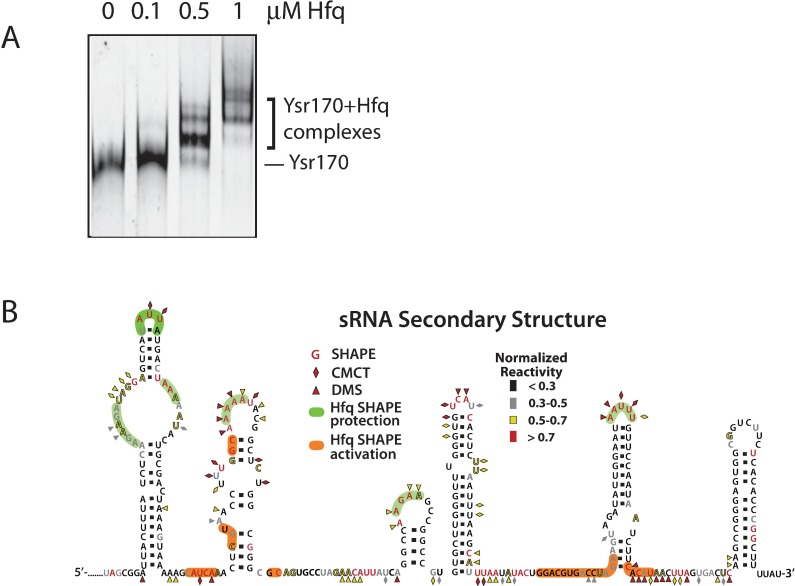
Chemical probing techniques were employed to determine Ysr170 secondary structure and interactions with Hfq. (A) An electrophoretic mobility shift assay (EMSA) was performed to evaluate Hfq-Ysr170 interactions. Ysr170 (80nM) was incubated with an equimolar concentration of Alexa Fluor 488-labeled deoxynucleotide primer, complementary to the Ysr170 3’-end, and increasing molar ratios (0–1μM) of Hfq hexameric protein, as determined by molecular weight. The RNA-protein complex was resolved on a native 6% polyacrylamide gel and analyzed using a Hitachi FMBio III imager. A representative of three independent experiments is shown. (B) Summary of probing data overlaid on the secondary structure. Ysr170 (0.5 μM) was folded in 1xHMK buffer containing 1.5 μM of Hfq at 37°C for 10 min. Range colors represent the relative normalized reactivity to each probe.[29] Colored nucleotides, diamonds, and triangles indicate SHAPE, CMCT, and DMS probing, respectively. Green and orange shading indicate regions in the structure where Hfq interactions lead to a decrease or increase in SHAPE reactivity, respectively.

We also examined the structural effects of Ysr170 interaction with the RNA chaperone Hfq, which has been shown to be essential for mediating sRNA/mRNA interactions in bacteria, including *Yersinia* species. (Fig 6) [28] To demonstrate direct Hfq-Ysr170 interaction, we performed an electrophoretic mobility shift assay (EMSA) and observed a band shift at 0.5 μM Hfq (~1:5 ratio of Ysr170 to Hfq), and multiple higher molecular weight bands at 1 μM Hfq. (Fig 6A) By SHAPE, Hfq interactions are observed in nearly all of the helices and in two junction regions of Ysr170, primarily in single-stranded RNA regions. (Fig 6B, green and orange) This data suggests that Hfq may interact at different sites on Ysr170 and as such, the higher order bands in the EMSA may represent multiple Hfq molecules binding to Ysr170. All together, this data suggests that Hfq provides structural stabilization to complex tertiary architecture in sRNAs, which is consistent with previous findings that Ysr170 expression is dependent on Hfq in *Yersinia*.[23]

### Structural modeling of Ysr170 interaction with gentamicin

**Fig 7:**
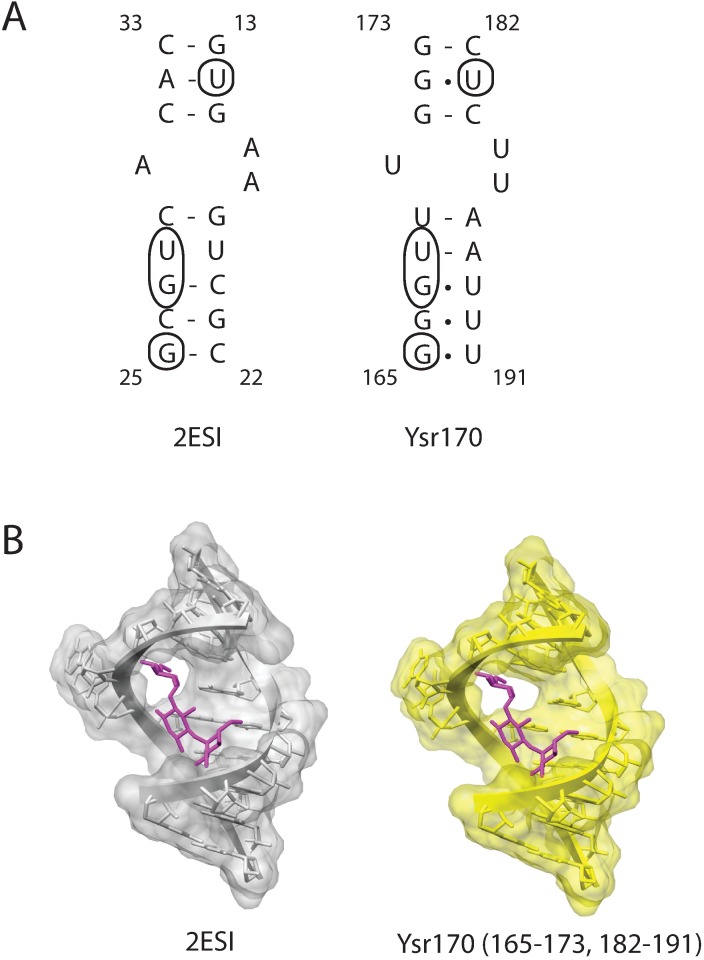
Structural modeling of Ysr170 binding to gentamycin. (A) The Ysr170 nucleotide region that binds to gentamycin, as determined by SHAPE analysis, is compared to the sequence of a structurally homologous region from 16S rRNA (2ESI). The circled nucleotides are identical between the 16S rRNA and Ysr170 sequences. (B) Modeled structures of gentamycin (purple) interaction with the 2ESI sequence and the Ysr170 binding site are depicted.

Our structural models support the stereochemical feasibility for gentamicin binding to Ysr170. The gentamycin binding site in Ysr170 was mapped to the fourth stem loop structure (residues 165–173 and 182–191) with a secondary structure that includes a duplex with a 1–2 nucleotide bulge. (Figs 5A and 7A) From the crystal structure (PDB accession code: 2ESI) of 16S rRNA, we know that three gentamycin molecules bind to RNA target sites. We chose gentamicin binding to a region that includes a 1–2 bulge as the template for the Ysr170 structural modeling. (Fig 7B) We developed a model that shows that gentamicin can bind to the Ysr170 target site in the same site and orientation as the gentamicin binding site in 16S rRNA. The encircled residues (Fig 7A) show key identical nucleotides that are conserved between the 16S rRNA-derived template and the Ysr170 target sequences.

## Discussion

sRNAs represent a primary layer of gene regulation that enables bacteria to adapt to changes in their immediate environment, including pathogen interactions with the host during infection. Consequently, sRNAs essential for regulation of virulence may be attractive targets for the development of novel therapeutic strategies against infectious disease. In particular, sRNA expression has been previously linked to *Yersinia* virulence. Differential sRNA expression in *Y*. *pestis* has been observed in response to temperature shift from 26°C to 37°C, which simulates host infection[10, 13], and in the lungs of infected mice.[14] The deletion of one sRNA candidate, ysr35, resulted in the attenuation of *Y*. *pestis* infection in a mouse model.[23] Another sRNA candidate, ysr141, was found to be expressed on the pCD1 plasmid, which encodes for T3SS effectors that are injected into the host cell during infection to subvert host immunity.[13] Ysr141 was found to target the 5’ untranslated region upstream of a T3SS effector, *yopJ*, to post-transcriptionally activate YopJ synthesis. Finally, the sRNA HmsB has been shown to enhance the activation of biofilm formation in *Y*. *pestis*.[30]

We compared our sRNA predictions to three previous *Y*. *pestis* RNA sequencing studies, including the identification of 31 sRNAs in *Y*. *pestis* KIM6+[10], 207 sRNAs in *Y*. *pestis* CO92[13], and 104 sRNAs in *Y*. *pestis* strain 201[14]. (Table 3) We observed ≥50% overlap in sRNA identification with the other three studies. We detected 17 out of the 31 sRNAs (55%) reported in the KIM6+ strain, including Ysr170. Of the 104 sRNAs identified in *Y*. *pestis* strain 201, we detected 81 sRNAs (78%), although 19 are likely part of 5’UTRs or operons and not included in our list of sRNAs in S2 Table. For *Y*. *pestis* CO92, 63 novel sRNAs were recently identified by comparing *Y*. *pestis* growth at 26°C and 37°C and another 144 sRNAs were found in *Y*. *pseudotuberculosis*.[23] We detected 102 of these combined 207 sRNAs (49%) and 19 are likely part of 5’UTRs or operons. While there can be significant overlap in sRNA identification between individual studies, other studies exhibited very little overlap [e.g. one sRNA was reported in common between [13] and [14]]. Differences in sRNA identification across independent studies are likely due to multiple factors, including different strain backgrounds, growth conditions, and sequence analysis methods. For example, different sequence read lengths were employed, ranging from 36 nt[13] to 75 nt[14] to 100 nt in this study.

**Table 3:** *Yersinia pestis* small RNA studies.

Study	Mapping strain	Biovar	Total sRNAs	Novel	Labels
Beauregard 2013	KIM 6+	Mediaevalis	31	14	Ysr151-187
Yan 2013	201	Microtus	104	78	sR001-104
Schiano 2014	CO92	Orientalis	207	63	Ysr1-150^1^ and Ysr188-Ysr251
This study	CO92	Orientalis	180	37	Ysr252-Ysr288

^1^150 previously identified in *Y*. *pseudotuberculosis* in Koo et al. 2011 and 144 confirmed in *Y*. *pestis*

Here, we have focused on Ysr170, a highly-expressed sRNA in *Y*. *pestis* that invaded THP-1 cells compared to pathogen that remained extracellular and was significantly upregulated at the human host temperature of 37°C compared to 26°C.[9] Our RACE and northern blot studies indicated that Ysr170 in *Y*. *pestis* CO92 consists of 362 nt that includes the shorter 125 nt transcript at the 3’ end previously identified in KIM6+.[10] In KIM6+, Ysr170 was also upregulated at 37°C and exhibited dependence on Hfq, consistent with our sequencing and SHAPE results in CO92. We note that the sRNA predictions in the KIM6+ study[10] were consistently shorter than all previously cited *Yersinia* sRNAs. For example, GlmZ is a 221 nt length sRNA[31], yet the KIM6+ study predicted a shorter transcript of 72 nt at the 3’ end.

From our study, we found that knockdown of *ysr170* expression significantly decreased infection efficiency of pathogenic *Yersinia* in cell culture, led to higher production of the pro-inflammatory cytokine TNF-α, and increased gene expression of IL-8 and the transcription factor EGR1, compared to infection with wild type *Yersinia* strains. These effects were mitigated in *Yersinia* cured of the pCD1 plasmid, suggesting that Ysr170 may regulate the host immune response through pCD1. Similar to other non-coding regulatory RNAs, it is likely that Ysr170 modulates expression of multiple downstream mRNA targets. Based on genome-wide transcriptomics analysis of a Ysr170 KD strain, Ysr170 appears to function as a global regulator of metabolic pathways and pathogenicity that enables *Yersinia* infection of the host. (Manuscript in preparation)

In addition to RNAseq, our lab has sought to utilize different methods to characterize sRNA structure and dynamics, including single molecule detection[9] and sRNA structural analysis by SHAPE in this current study. SHAPE has been employed to analyze RNA secondary structure and RNA binding interactions for riboswitches[32], mRNAs[33], and long non-coding RNAs[34]. These approaches can enable a more comprehensive understanding of sRNA function and can highlight potential interactions with small molecule inhibitors to block sRNA folding or binding to target mRNAs. In the SHAPE analysis of Ysr170 binding to different antibiotics, we observed that only gentamicin, out of six tested antibiotics, exhibited significant binding to Ysr170. A second antibiotic, kanamycin, displayed a weaker interaction. Interestingly, gentamycin and kanamycin bound to Ysr170 at independent internal stem-loop sites, indicating differential binding affinities based on RNA structure. Classical aminoglycosides such as gentamicin are potent antibiotics that bind to the decoding A-site of ribosomal RNA and cause errors in protein translation and premature termination.[35] From SHAPE, we observed that gentamicin stabilized Ysr170 helix 4, which contains a bulge that structurally resembles its ribosomal binding site, suggesting that antibiotics are capable of binding to structurally conserved RNA. We also developed a structural model that demonstrates the feasibility of gentamicin binding to its target site in Ysr170.

Although it is not yet clear whether aminoglycoside binding to sRNAs is physiologically relevant, there is precedence for aminoglycoside interactions with non-ribosomal RNA species. Based on examination of aminoglycoside binding to the hammerhead ribozyme, the positively charged amino groups of the aminoglycoside are thought to form electrostatic interactions with the negatively charged phosphate backbone of the nucleic acids.[36] Aminoglycosides have also been shown to bind to various types of RNA, including the influenza A virus RNA promoter[37], the HIV Rev RNA recognition element[38] and TAR RNA[39], and thymidylate synthase mRNA[40]. These molecules have prominent internal loops and bulges, as measured by a variety of biophysical and spectroscopy methods, which is consistent with our study that demonstrates aminoglycoside binding to internal stem loops in Ysr170 by SHAPE.

Based on our SHAPE studies with Hfq, we found that multiple sites in Ysr170 exhibited modified reactivity in response to Hfq binding, suggesting that Ysr170 undergoes global structural changes upon interaction with the chaperone. Hfq is a global post-transcriptional regulator that acts by mediating interactions between many sRNAs and their cognate mRNA targets. Hfq has been shown to be a key regulator in *Y*. *pestis* stress response, intracellular survival and pathogenesis, presumably by regulating expression of stress and virulence genes via interactions with specific sRNAs.[41] Our results are consistent with other studies that demonstrate extensive structural changes in sRNAs upon Hfq binding, such as the *E*. *coli* sRNAs OxyS and RprA[42], the *Vibrio cholera* sRNA Qrr1[43], and the *E*. *coli* sRNA DsrA[44] by small-angle scattering. This structural flexibility in sRNAs likely facilitates base pairing with different target mRNAs in conjunction with the RNA chaperone function inherent to Hfq.

Altogether, our studies provide insight into the function and structure of Ysr170 in pathogenic *Yersinia*. Using SHAPE, we have also characterized a variety of secondary structures for additional sRNAs identified in this study (data not shown), indicating that sRNAs exhibit a broad range of folding architectures, similar to proteins, that dictate their binding interactions. Thus, it is likely that the spatial organization of sRNA secondary and tertiary folding will ultimately determine their functions. Understanding this structure-function correlation will provide guidelines for developing novel therapeutics that specifically target bacterial sRNAs. We expect that development of small molecules to inhibit key sRNAs that function in virulence can provide an alternative strategy that is complementary to standard antibiotics to combat infectious disease.

## Supporting Information

S1 Fig Expression plots of analyzed sRNAs. For each sRNA prediction, we plotted the relative coverage (reads per billion) in a 3,000 bp window and a larger 10,000 bp window to better detect expression across operons. For the detection step only, we combined replicates and used a single black dotted line for control and solid red or blue lines for intracellular and extracellular conditions, respectively. We included a number of genome features in the plots including protein coding regions and pseudogenes from RefSeq, ERIC and YPAL repeats[16], computational predictions of sRNAs using SIPHT[17], and rho-independent transcription terminators using TransTermHP.[18] The CDSs are marked in green, pseudogenes in gray, repeats in yellow, putative sRNAs in red, and terminators in black.
(PDF)

S1 Table Primers and probes used in this study. This list contains the primer and probes used for PCR and RACE analysis and the homology of *Y*. *pestis* sRNAs identified by RACE to other *Yersinia* species.
(DOCX)

S2 Table sRNA predictions from transcriptomics analysis. This is a list of 180 small RNAs detected and the DEseq results table with mean normalized counts, fold change and p-values.
(XLSX)
