# Supplementary figures and images for: Functional and Structural Analysis of a Highly-Expressed Yersinia pestis Small RNA following Infection of Cultured Macrophages
Source: PLoS One. 2016 Dec 28;11(12):e0168915. doi: 10.1371/journal.pone.0168915 (PMC5193452; doi:10.1371/journal.pone.0168915)

S1 Figure. Expression plots of analyzed sRNAs.

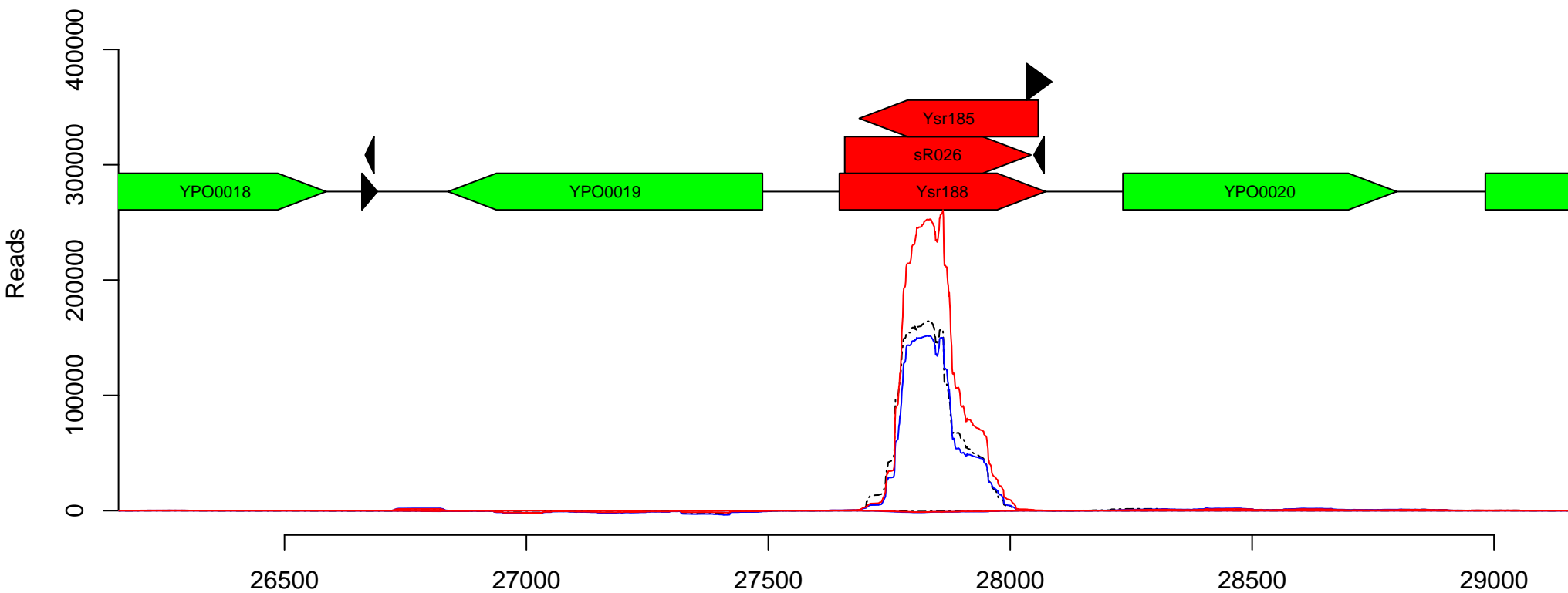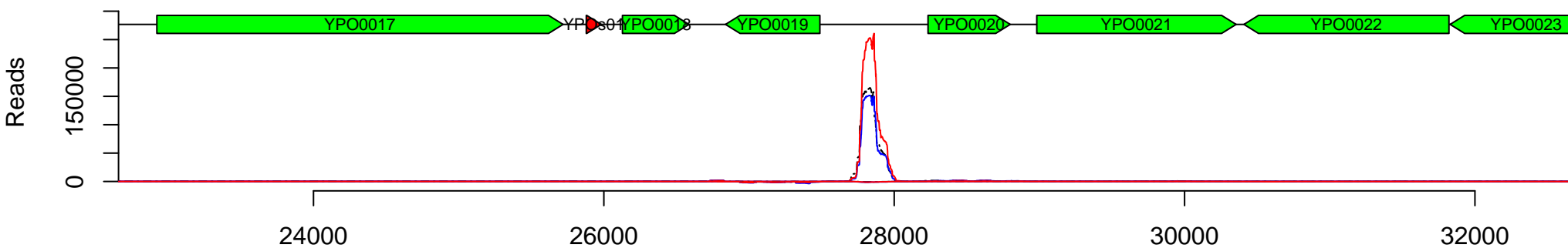

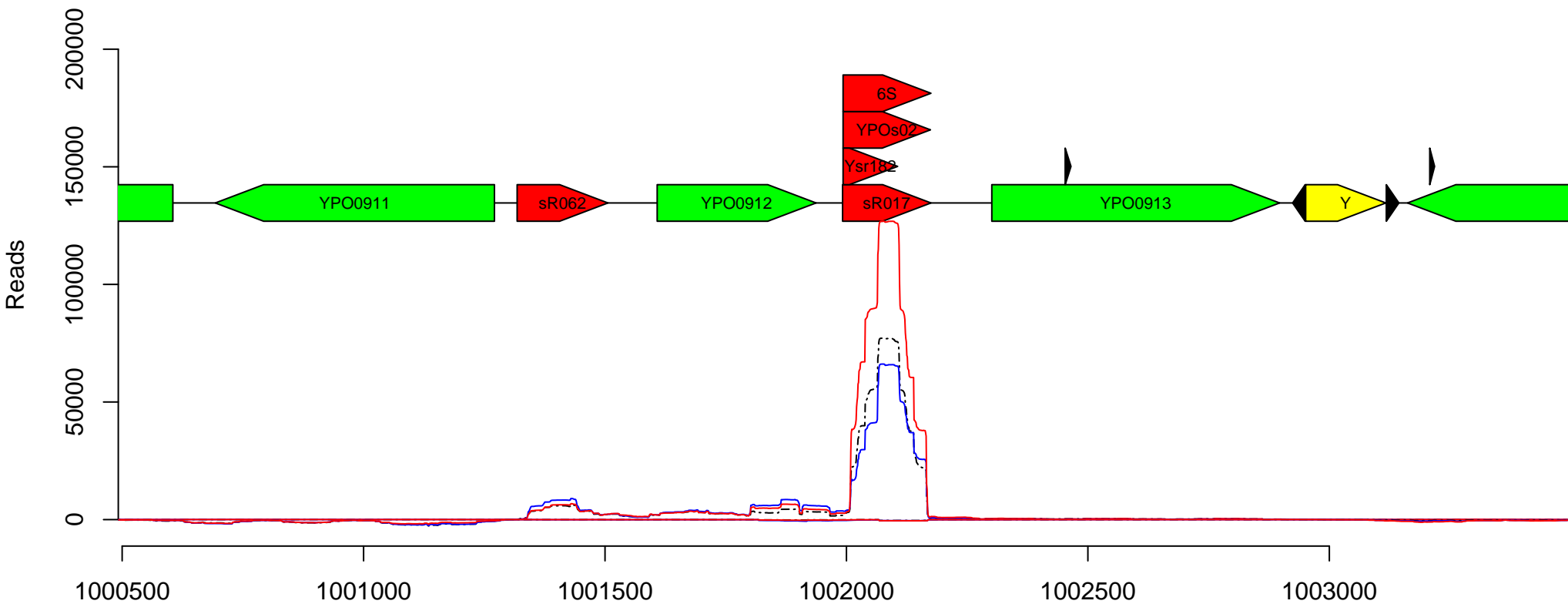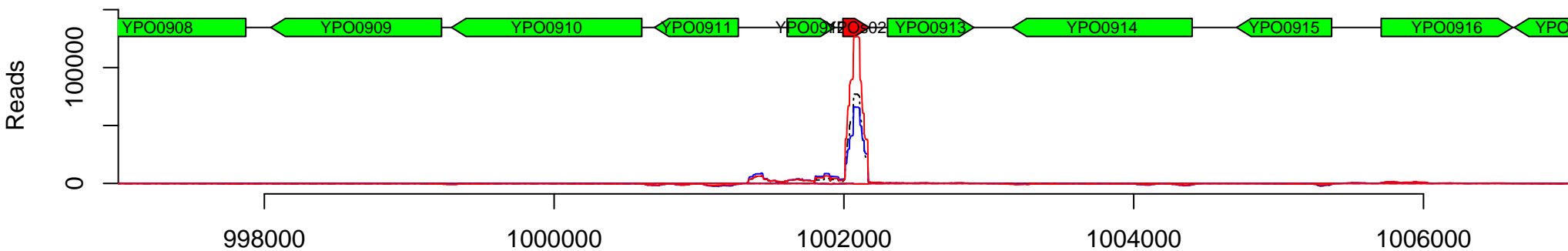

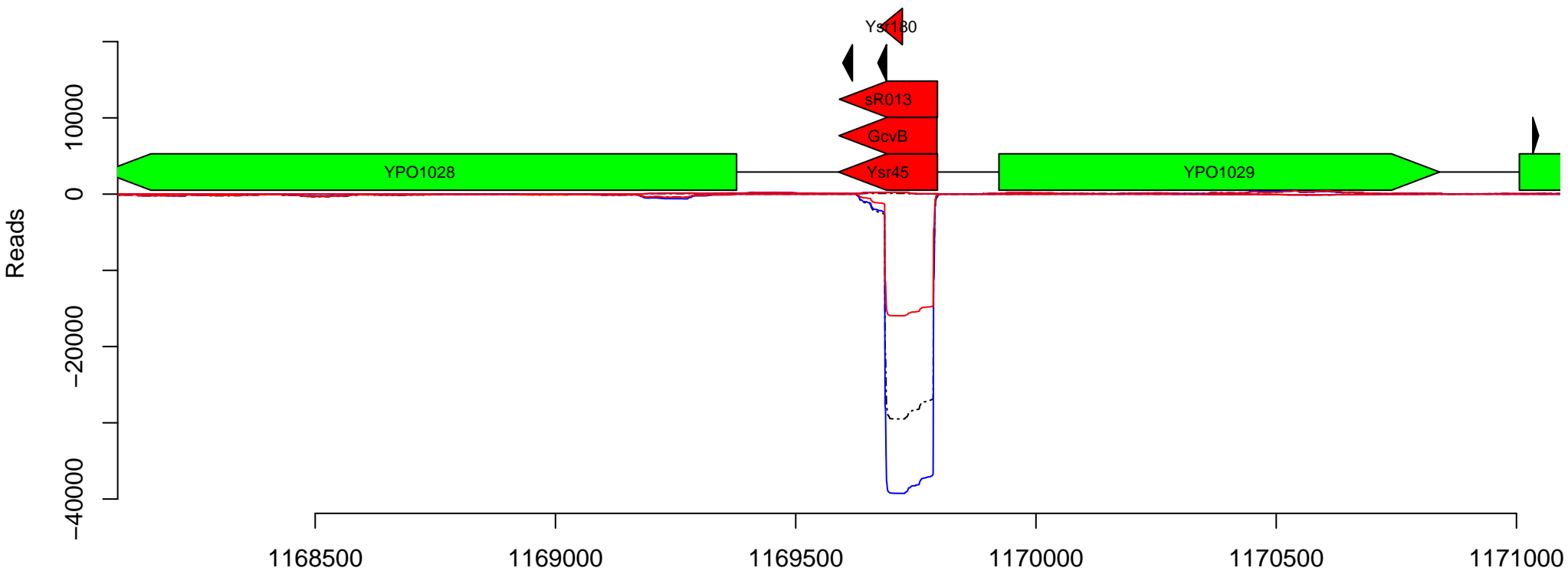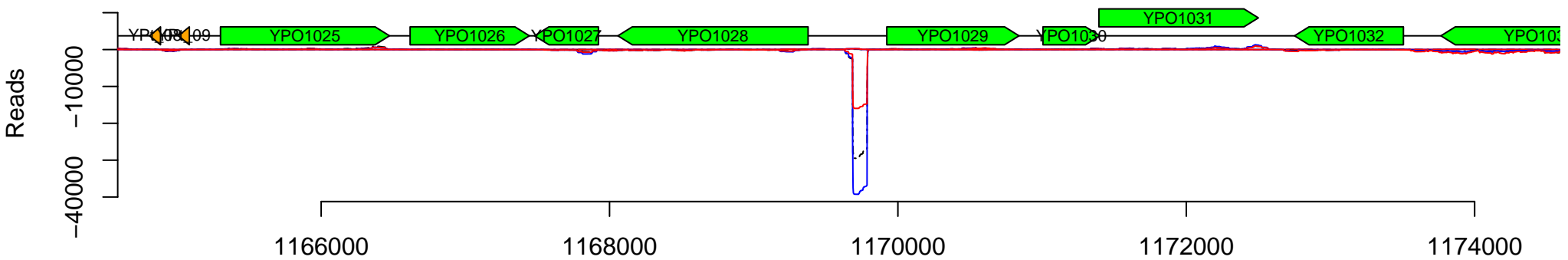

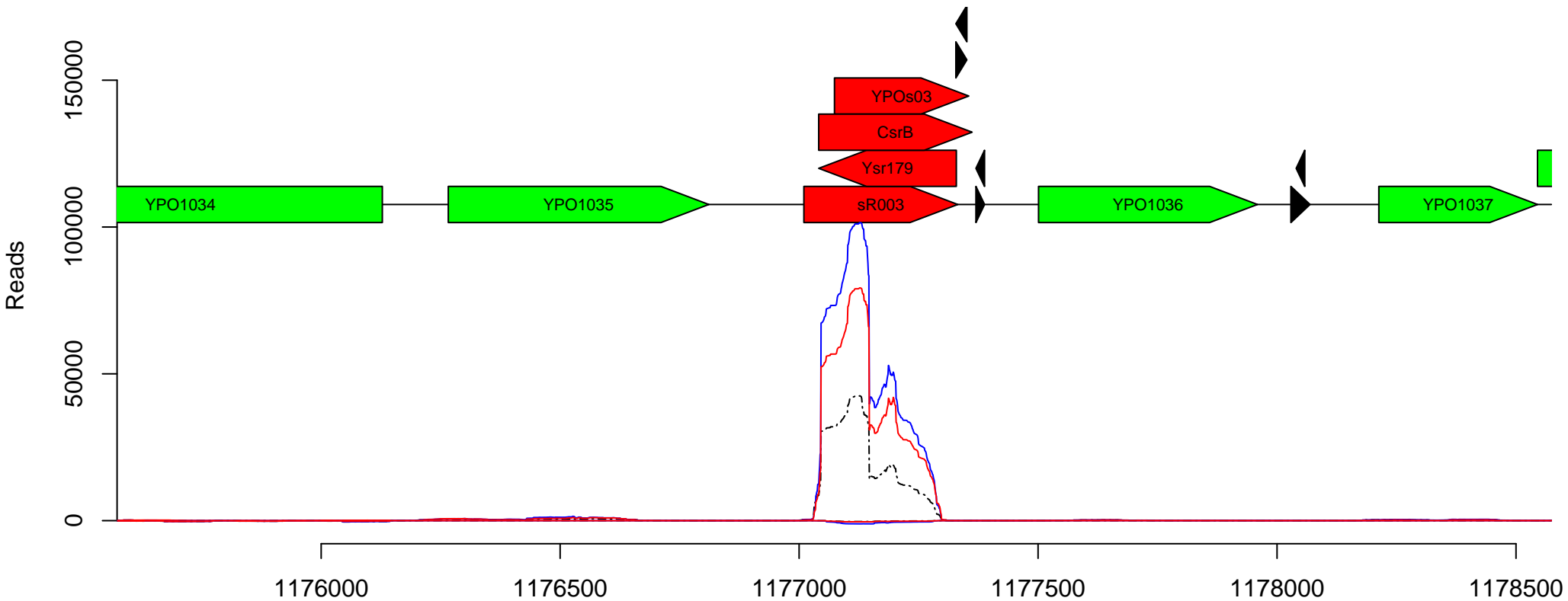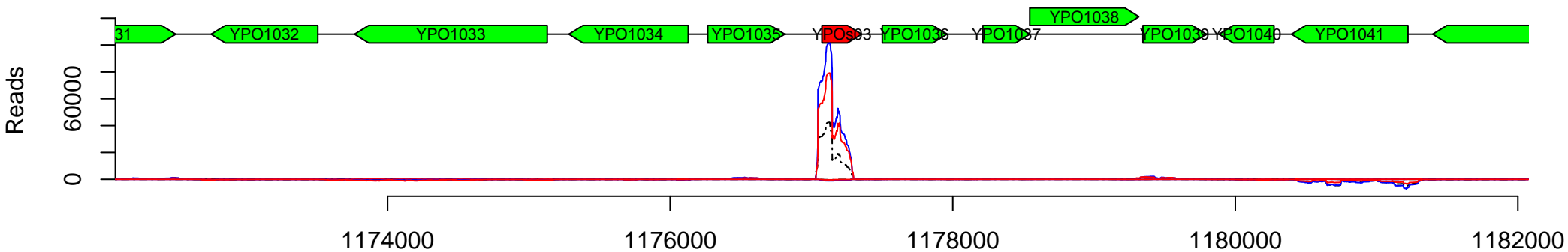



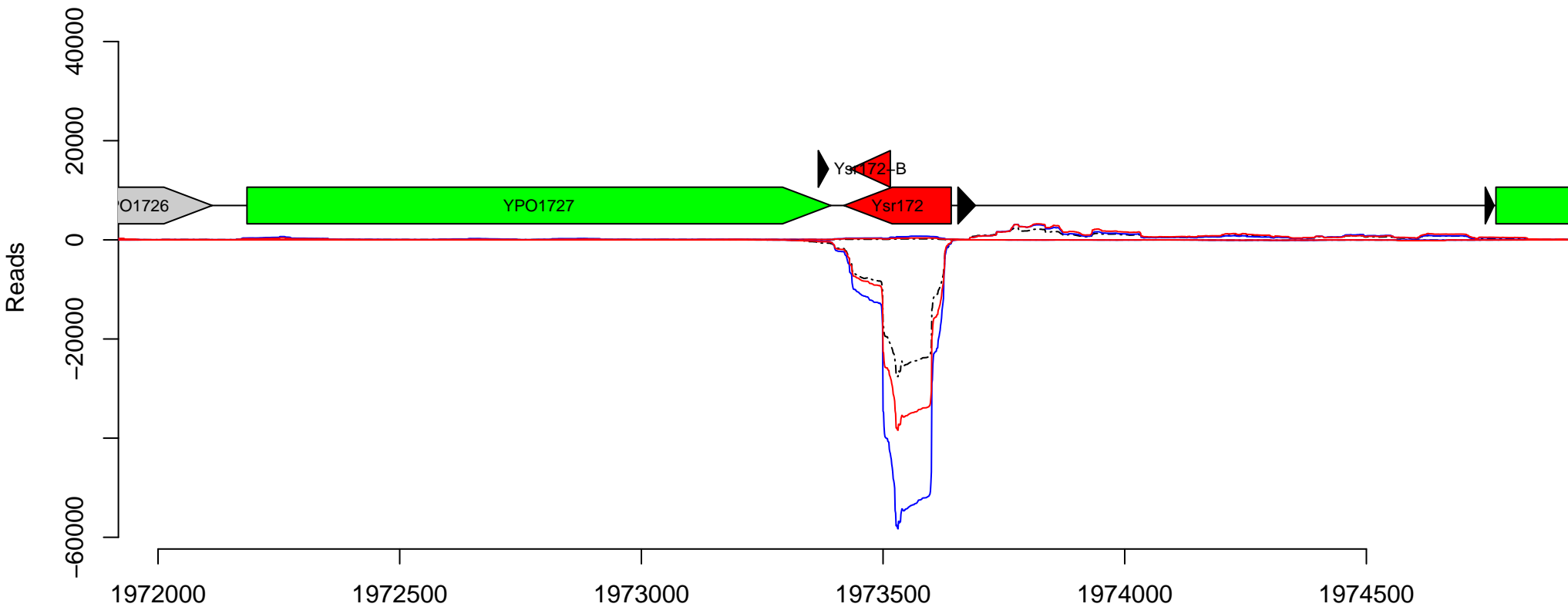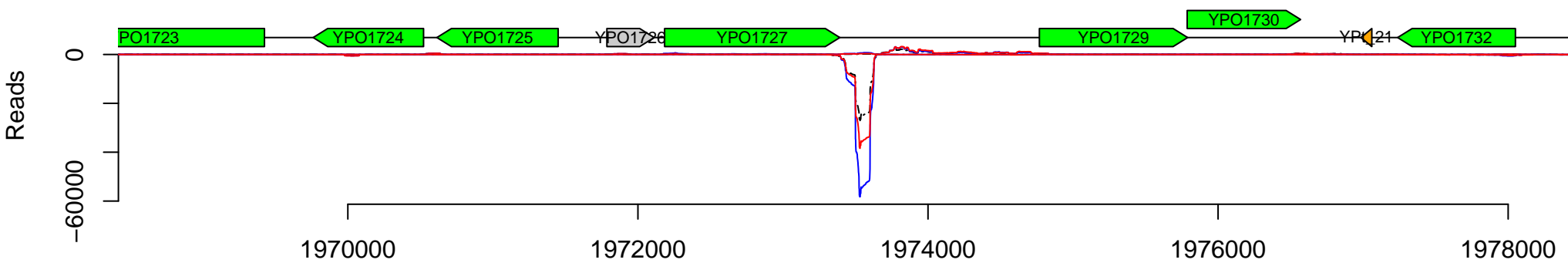

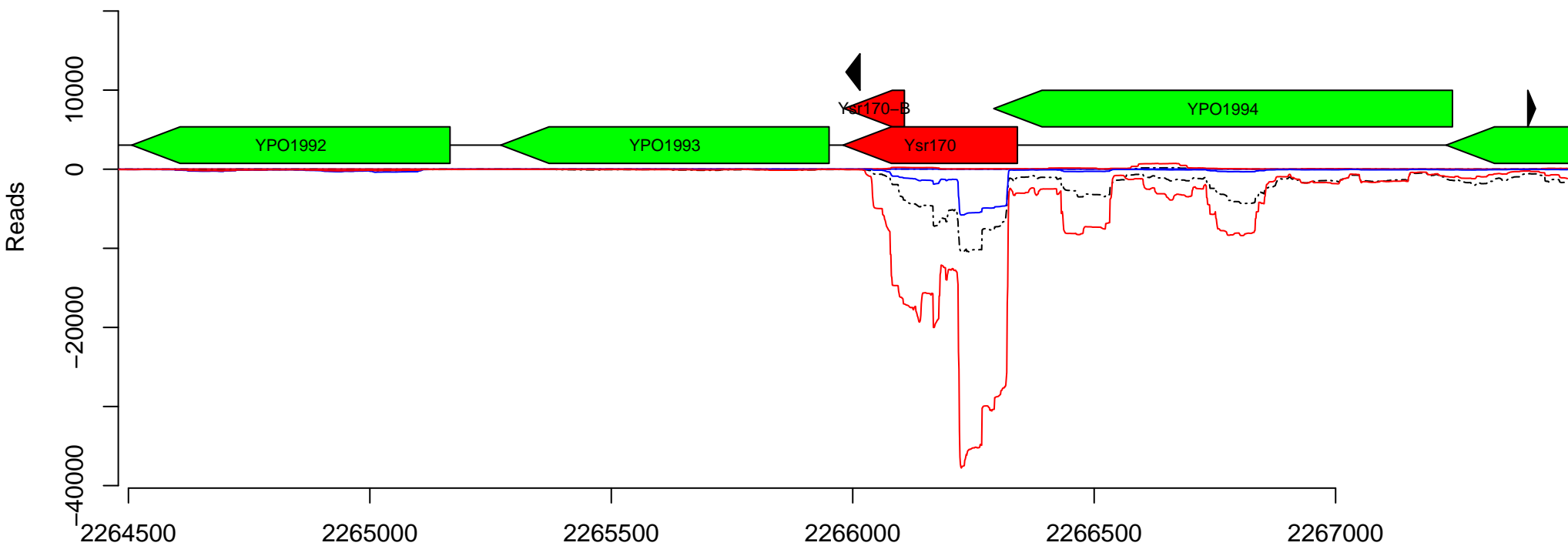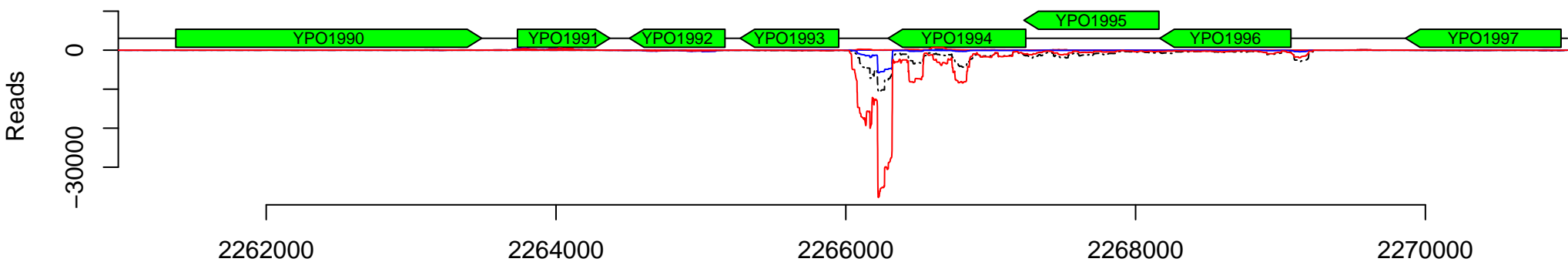

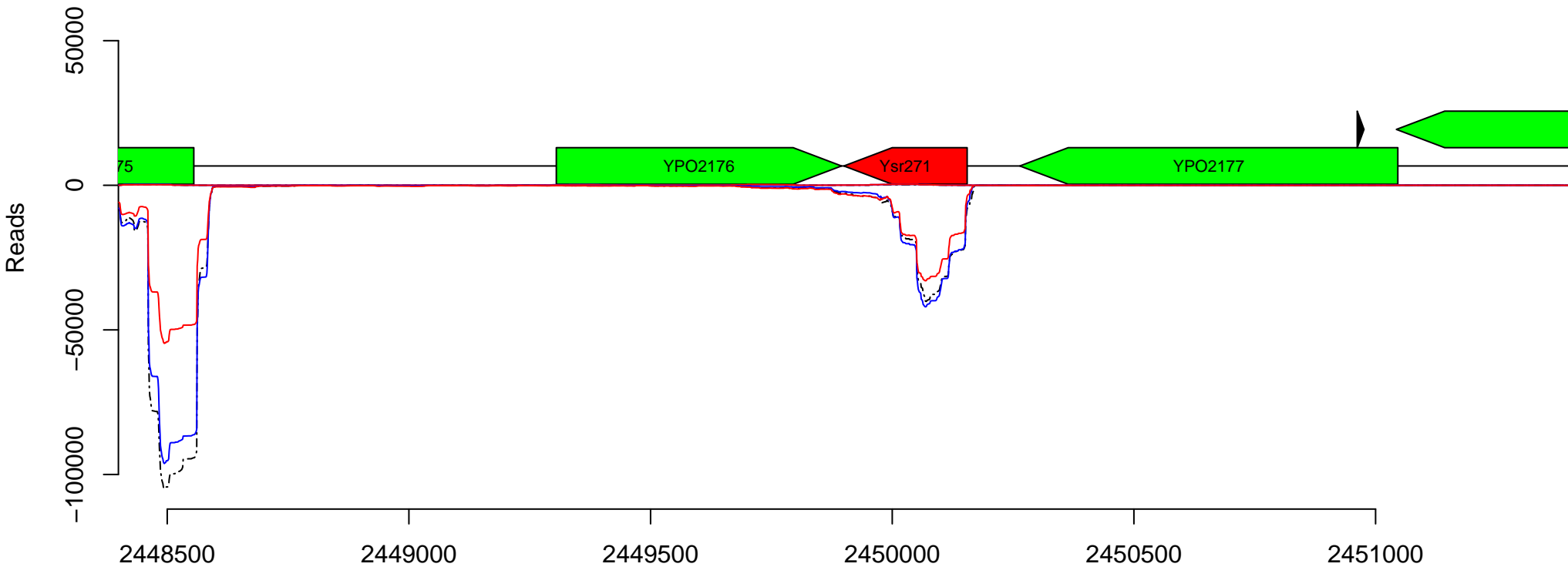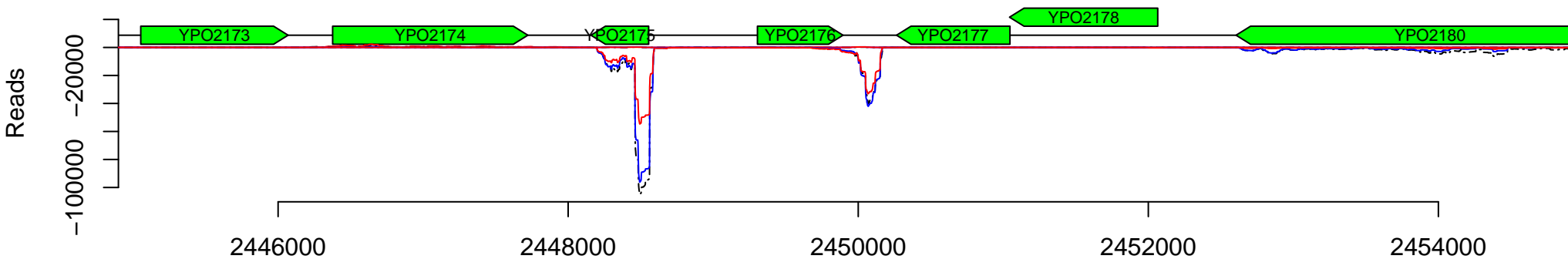

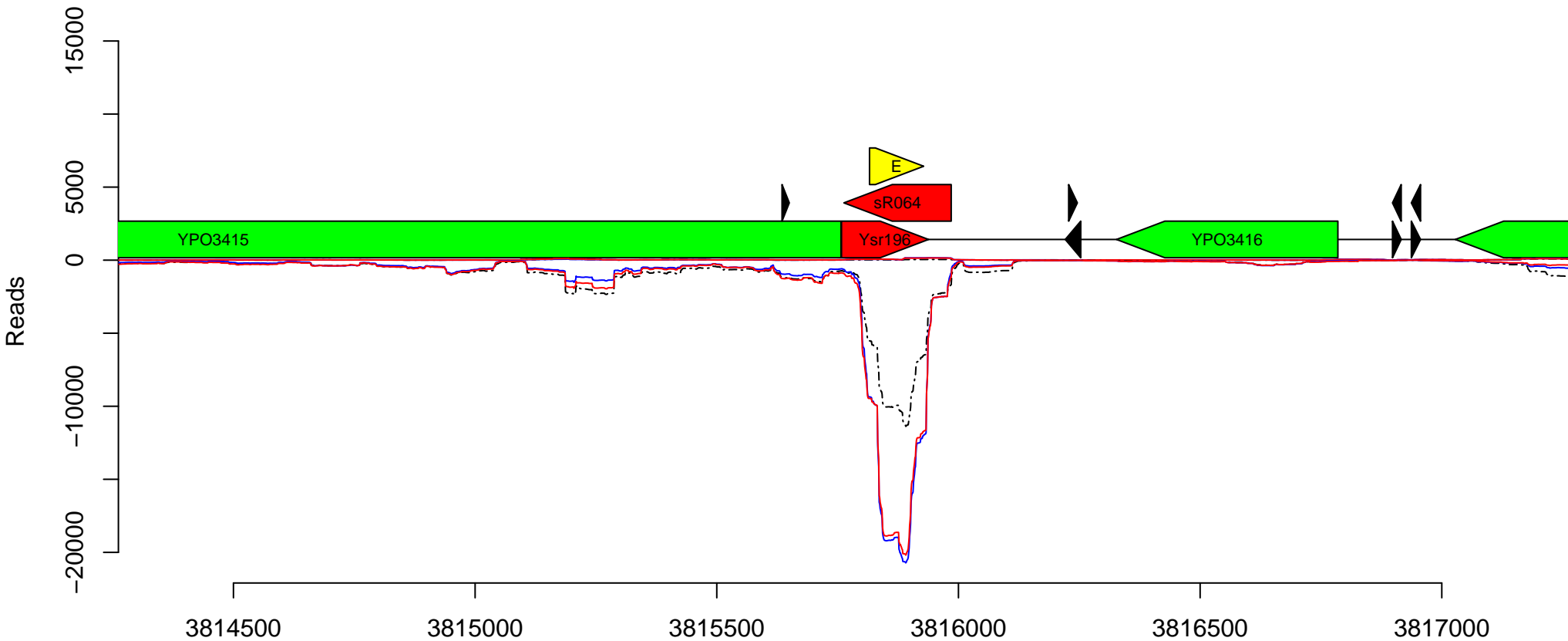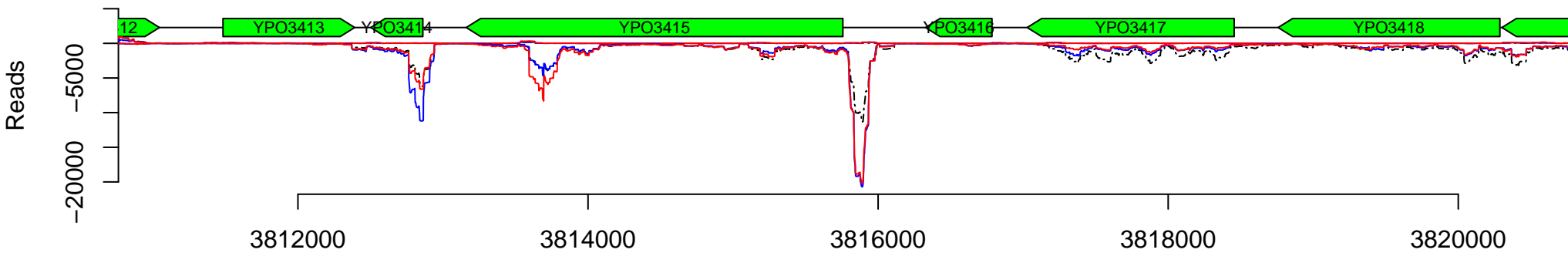

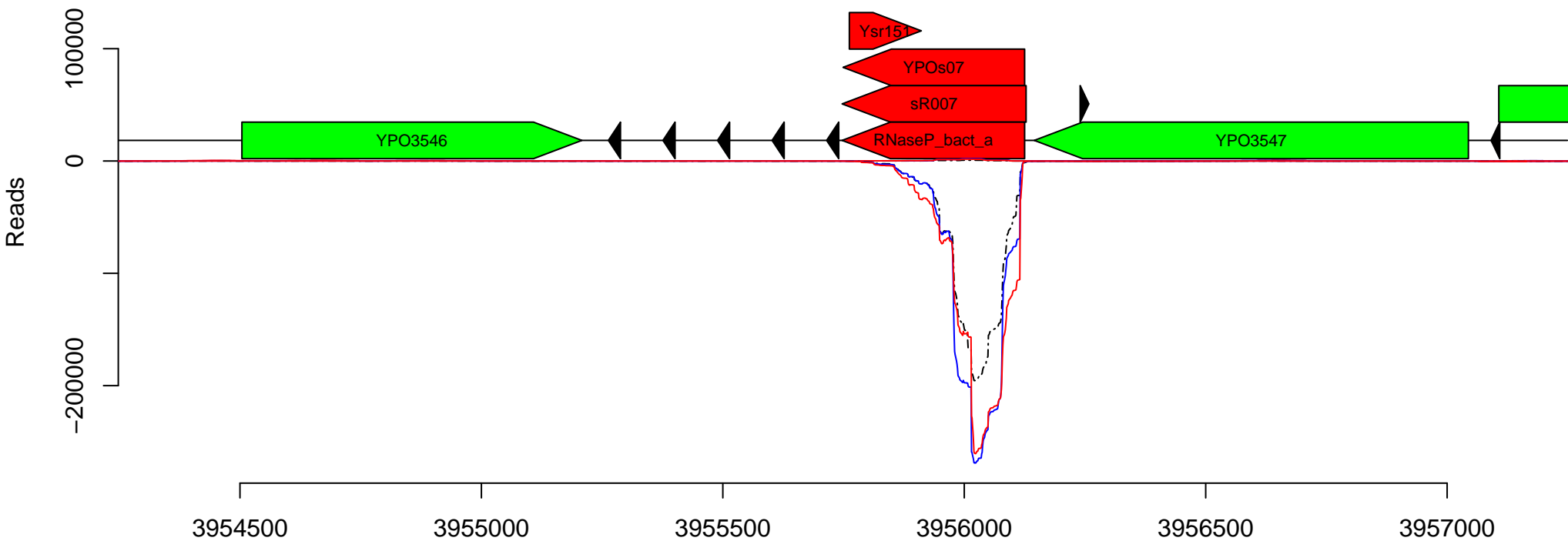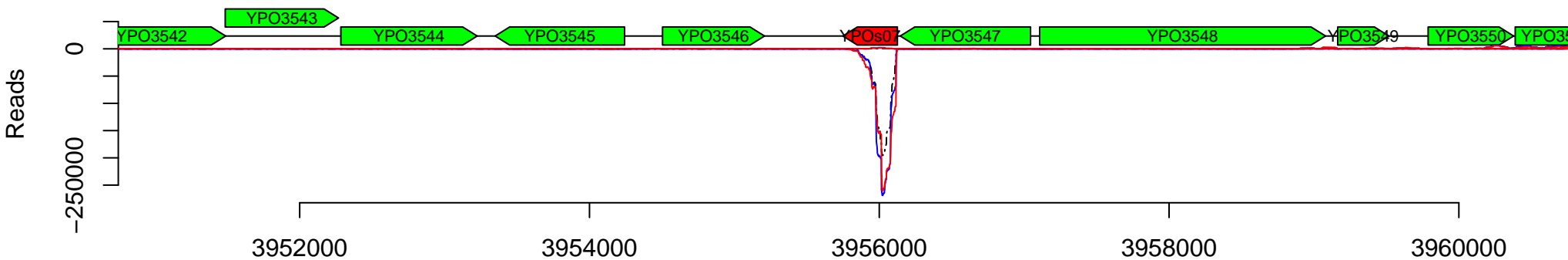

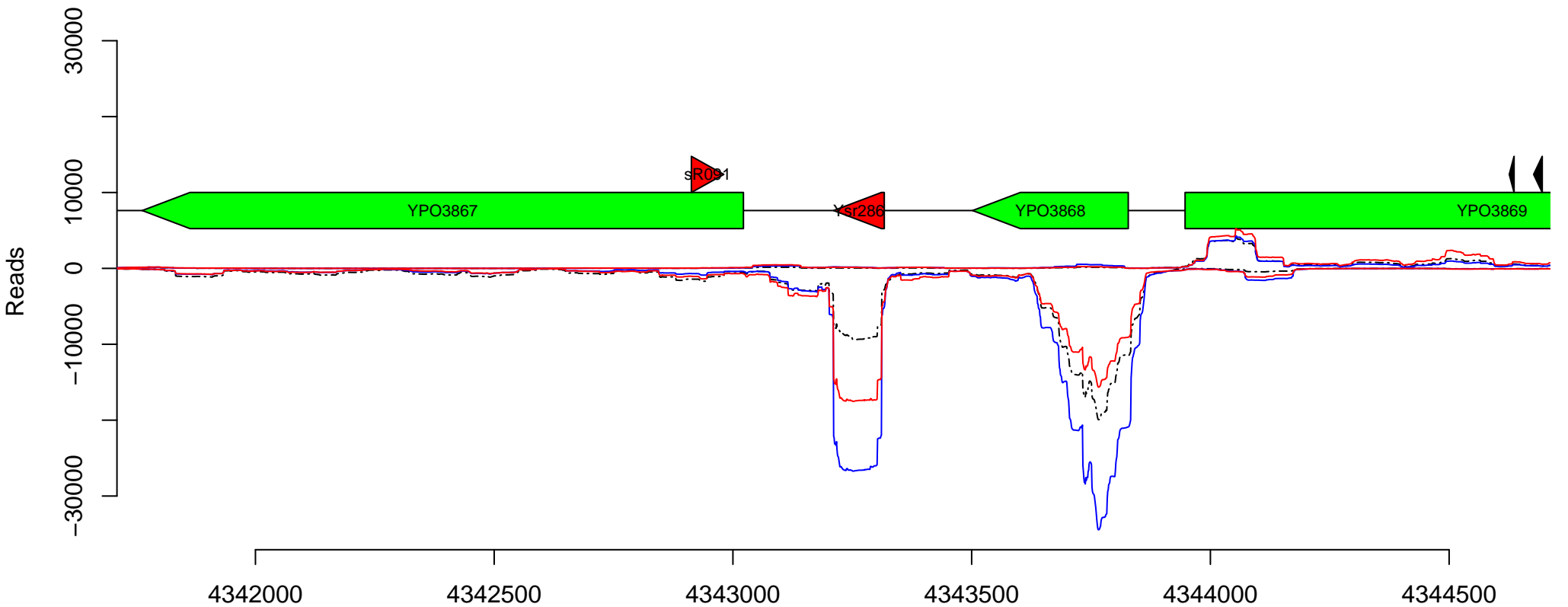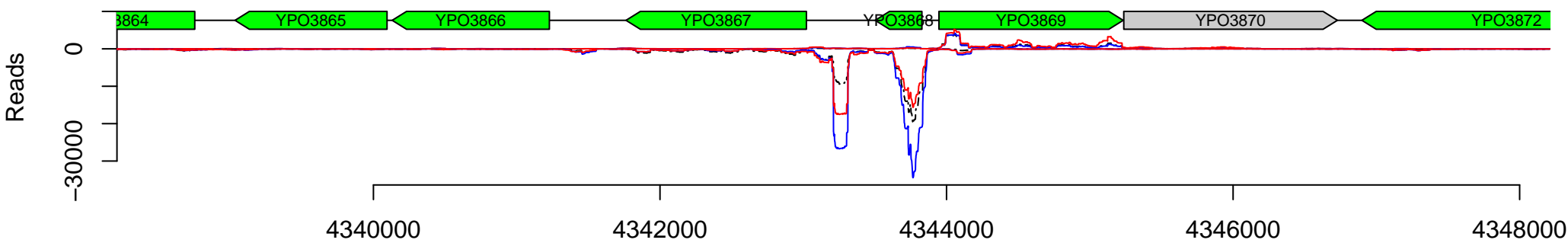

Supplement: S1 Fig — For each sRNA prediction, we plotted the relative coverage (reads per billion) in a 3,000 bp window and a larger 10,000 bp window to better detect expression across operons. For the detection step only, we combined replicates and used a single black dotted line for control and solid red or blue lines for intracellular and extracellular conditions, respectively. We included a number of genome features in the plots including protein coding regions and pseudogenes from RefSeq, ERIC and YPAL repeats[16], computational predictions of sRNAs using SIPHT[17], and rho-independent transcription terminators using TransTermHP.[18] The CDSs are marked in green, pseudogenes in gray, repeats in yellow, putative sRNAs in red, and terminators in black. (PDF) [file pone.0168915.s001.pdf]
