# Supplementary material for: Functional and Structural Analysis of a Highly-Expressed Yersinia pestis Small RNA following Infection of Cultured Macrophages
Source: PLoS One. 2016 Dec 28;11(12):e0168915. doi: 10.1371/journal.pone.0168915 (PMC5193452; doi:10.1371/journal.pone.0168915)
Supplement: S1 Table — This list contains the primer and probes used for PCR and RACE analysis and the homology of Y. pestis sRNAs identified by RACE to other Yersinia species. (DOCX) [file pone.0168915.s002.docx]

**S1 Table:** **Primers and probes used in this study and RACE sequences**

| **Northern blotting probes** | | |
| --- | --- | --- |
| Oligo dT | 5’-/5biosg/T_20_-3’ | |
| 5s rRNA | 5’-/5biosg/ACACTTGTCAGCCAGTCAAC-3’ | |
| sR073 | 5’-/5biosg/GAAATCATCATCTACCAGCGCAGC-3’ | |
| Ysr28 | 5’-/5biosg/GGTGATTATTATCCCACCGTGCG-3’ | |
| Ysr72 | 5’-/5biosg/CACGTGTTTTAGCGACAGACTCAC-3’ | |
| Ysr99 | 5’-/5biosg/GCATAACGAAATAGCCCTTGCTCC-3’ | |
| Ysr100 | 5’-/5biosg/GATGCATATGTAGGGGTACAGCAG-3’ | |
| Ysr104 | 5’-/5biosg/GAATGAAGGGTCTACTTCCCCTAC-3’ | |
| Ysr114 | 5’-/5biosg/CTGCTTTGTTGCTGGATATCCAC-3’ | |
| Ysr165 | 5’-/5biosg/CTAATGCCACTCGCTTAACTGTGC-3’ | |
| Ysr170 | 5’-/5biosg/AGTGATGACACCCAAACCCAAG-3’ | |
| Ysr172 | 5’-/5biosg/GCTGCCAGCGTTATTCAGTATTCG-3’ | |
| Ysr260 | 5’-/5biosg/CTTCTGGAGTGACAGCCCAAATAG-3’ | |
| Ysr273 | 5’-/5biosg/CCTTACAAAGCATTTCACGGTGC-3’ | |
| Ysr277 | 5’-/5biosg/GTTTGACTCCGTCAGGTGATACG-3’ | |
| Ysr283 | 5’-/5biosg/GAAATAACAAAGACGCCGTGCGC-3’ | |
| Ysr283 | 5’-/5biosg/GAAATAACAAAGACGCCGTGAGC-3’ | |
|  | | |
| **Cloning of ysr170 and Hfq** | | |
| 170senF | 5’-TTTCTCACAGTATTGGAAGCATGCC-3’ | |
| 170senR | 5’-ATAAAAGCCGGGTGTGAGAAGACC-3’ | |
| HfqF | 5’-GATCGGATCCGAATGGCTAAGGGGCAATCTTTGCAA-3’ | |
| HfqR | 5’-GATCAAGCTTTTATTCAGCGTCATCACTGTCCTG-3’ | |
|  | | |
| **RACE primers** | | |
| 72 RACE-F | | 5’-GTGAGTCTGTCGCTAAAACACGTG-3’ |
| 72 RACE-R | | 5’-TCGCCAAACCCAGTGTGATACG-3’ |
| 114 RACE-F | | 5’-GCCGCATGCCAAAATGAAAATAAC-3’ |
| 114 RACE-R | | 5’-GACCATCACAAACTCAATTTTTTTCAC-3’ |
| 165 RACE-F | | 5’-GCACAGTTAAGCGAGTGGCATTAG-3’ |
| 165 RACE-R | | 5’-AGCCCATTTAGCTGGCTGAACG-3’ |
| 170 RACE-F | | 5’-CTTGGGTTTGGGTGTCATCACTC-3’ |
| 170 RACE-R | | 5’-CACACGGCCGGCTTCTTG-3’ |
| 283 RACE-F | | 5’-GCTCACGGCGTCTTTGTTATTTC-3’ |
| 283 RACE-R | | 5’-GCCTTTATGAGGGCTCGAAC-3’ |
|  | | |
| **Real-time PCR primers** | | |
| 114PCR-F | 5’-TGAAAATAATTACGCTTGTAAAAACA-3’ | |
| 114PCR-R | 5’-TTATCTGCTTTGTTGCTGGA-3’ | |
| 170PCR-F | 5’-AACCTTTGGCAAAAATACGG-3’ | |
| 170PCR-R | 5’-TTAAGAGTGATGACACCCAAACC-3’ | |
| 172PCR-F | 5’-GCCTGAGCGATCACCTTTAAT-3’ | |
| 172PCR-R | 5’-ACAAGCGGCGAATACTGAAT-3’ | |
| 283PCR-F | 5’-CAAACCCCGATAATTCGATCT-3’ | |
| 283PCR-R | 5’-GCAGCGGGAGAAGAGAGAAA-3’ | |
|  | | |
| **RACE sequences** | | |
| **Ysr72** (2348534-2348614) 81nt  AGTTAATTATTCTAACTTCATGAGGGTTTTAGGCGTATCACACTGGGTTTGGCGAGTGAGTCTGTCGCTAAAACACGTGTA  ***Homology:*** 100% to *Y. pseudotuberculosis* | | |
| **Ysr114** (1543770-1543972) 203nt  GTCATGACCCGAGGTGAAAATAATTACGCTTGTAAAAACAATTGCATTTGTGAAAAAAATTGAGTTTGTGATGGTCGCCGCATGCCAAAATGAAAATAACGGCTTGATTGTTTTTTATTGGAATGTGGATATCCAGCAACAAAGCAGATAAAAGCGGGAAAACAGATAAAAAAGCGGGTGGCCCATGTAGCCACCCGTATAGA  ***Homology:*** 100% to *Y. pseudotuberculosis* | | |
| **Ysr165** (1846560-1846676) 117nt  GTACCAAGAACCCGCGGAGATAGTGTTAGTTCTGATCGTTTAGCCAGAAAGAGCGTTCAGCCAGCTAAATGGGCTGCACAGTTAAGCGAGTGGCATTAGCATTAACGCGGGTTTCTC  ***Homology:*** ~99-100% to *Y. pseudotuberculosis* | | |
| **Ysr170** (2266341-2265980) 362nt  TTTCTCACAGTATTGGAAGCATGCCATGCATGAGTCCAAAACCCCGCTAATATAGTTAGCGGATTACTTTATCTCAAGAAGATAGGAGTCATATTATGACTAAAAATACTGCGACTAAAGTAAAAAGCATCAAACTCGTAACCTTTGGCAAAAATACGGCTCTGGCGGGCGCAGTGCCTAGAACATTATCAGGCCAAGAAGCCGGCCGTGTGCTTGGGTTTGGGTGTCATCACTCTTAATTTAAGCATTTAATATACTGGACGTGACCTAGAAGTAGATATTGGAATAATTTGTTCCAATAATCTTCACATAACTTAGTGACTCAAGCCGGGAGTGGCGGTCTTCTCACACCCGGCTTTTAT  ***Homology:*** 100% to *Y. pseudotuberculosis* | | |
| **Ysr283** (4122174-4122369) 196nt  GGTACCAGACTGCTGACAAACCCCGATAATTCGATCTTAAATGGTGAGGATAGGCAGGGAAGGCGCGGTTCGAGCCCTCATAAAGGCGCTCACGGCGTCTTTGTTATTTCTCTCTTCTCCCGCTGCAGGCACGTTGTCATCAACCTCGGTACCTTCCTTCTTCGCCTCCCTTTGATGGGTGGCATCATCTCCTCAG  ***Homology:*** ~99% to *Y. pseudotuberculosis* | | |
